# Transport-induced voltage penalties of aqueous electrochemical reactions involving proton or hydroxide transfer in non-extreme pH conditions

**DOI:** 10.1039/d6ey00240d

**Published:** 2026-09-23

**Authors:** Gerard Prats Vergel, Yvette Ziggers, Min Li, David A. Vermaas, Thomas Burdyny

**Affiliations:** a Department of Chemical Engineering, Faculty of Applied Sciences, Delft University of Technology van der Maasweg 9 2629 HZ Delft The Netherlands T.E.Burdyny@tudelft.nl; b e-Refinery Institute, Delft University of Technology Leeghwaterstraat 39 2628 CB Delft The Netherlands

## Abstract

Electrolyzers operating in non-extreme pH conditions are gaining interest as they offer advantages in stability and compatibility with different feedstocks, reactants and products. However, the cell voltages reported are often substantially higher than their thermodynamic equilibrium potential, undermining their energy efficiency and scalability. Here, we show that this discrepancy originates from ionic transport through the cell and applies to any electrochemical reaction involving H^+^ or OH^−^, such as water, CO_2_ and nitrate reduction or water oxidation. These transport-induced penalties are usually not accounted for until much later in the development process. While strongly acidic and alkaline electrolyzers can sustain current while maintaining pH symmetry between the electrodes, non-extreme pH electrolyzers inevitably develop local large pH gradients that lead to asymmetric conditions. This local pH asymmetry generates concentration overpotentials that can reach 0.5–1 V depending on the applied current density and temperature. Finally, we discuss strategies to minimize local pH shifts, assess their risks for industrial implementation, and identify pathways to reduce the transport-induced pH-asymmetry voltage penalties of non-extreme pH electrolyzers.

## Abstract 2

Electrolyzers operating in non-extreme pH conditions suffer from large concentration overpotentials due to local asymmetry between electrodes.

## Graphical abstract

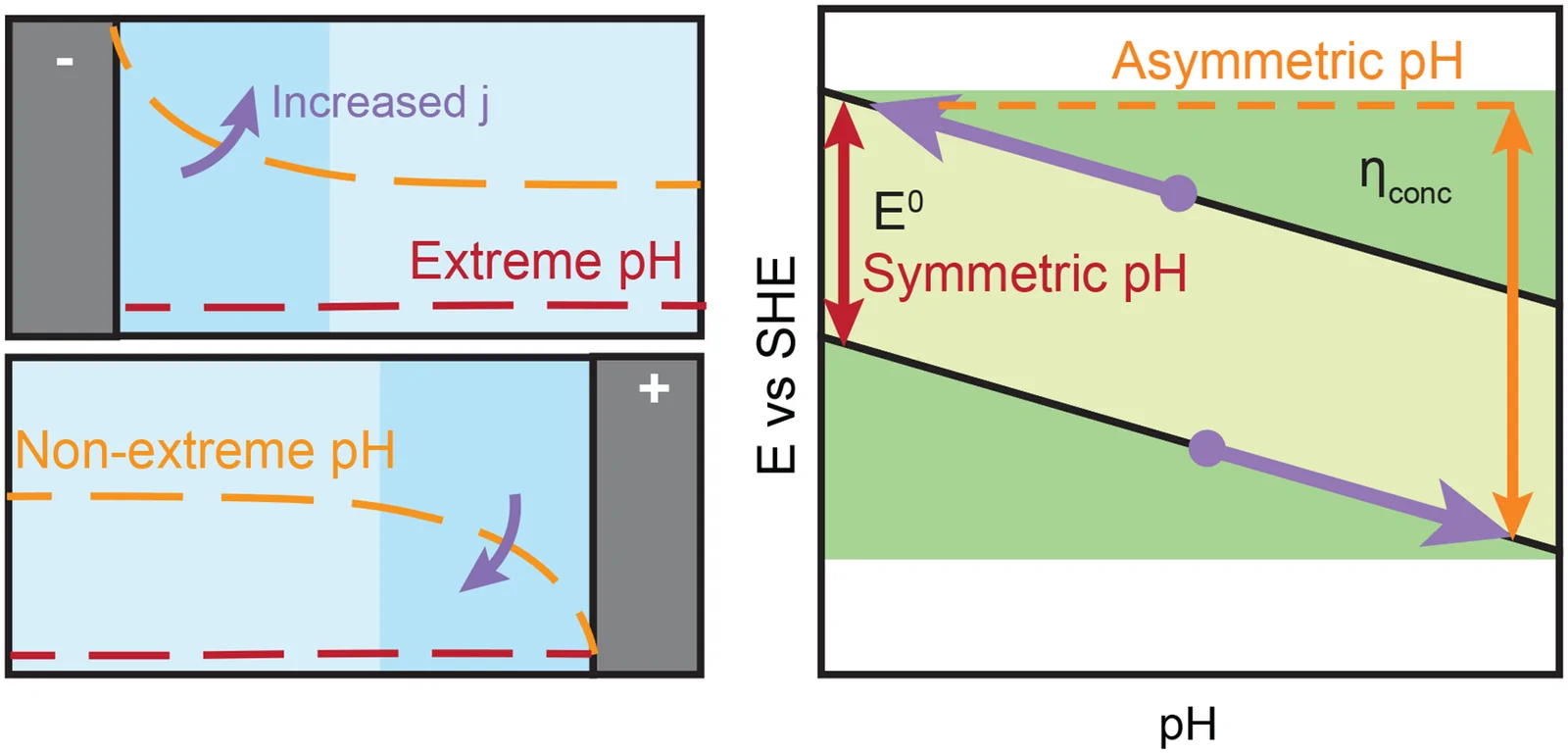

**Broader context**

Electrolysis enables the conversion of abundant feedstocks into valuable chemicals and fuels using renewable electricity. As emerging electrochemical technologies transition from catalyst discovery toward engineered membrane-electrode assembly systems, minimizing cell voltage becomes increasingly important because electricity dominates operating costs. Many promising reactions, including CO_2_ and nitrate electrolysis, necessarily operate under non-extreme pH conditions to maintain catalyst performance, product selectivity, or feedstock compatibility. Here we show that aqueous electrochemical reactions involving H^+^ or OH^−^ transfer share a common but underappreciated limitation: sustained current inevitably generates local pH asymmetry between the cathode and anode, leading to concentration overpotentials of up to 0.5–1 V. Rather than treating these voltage penalties as reaction-specific phenomena, we establish a general thermodynamic and transport framework that explains their origin across water, CO_2_, and nitrate electrolysis, and more broadly across aqueous electrochemical conversion technologies. By providing this framework, our work enables these intrinsic voltage penalties to be recognized during the early stages of technology development, helping guide reaction selection, electrolyte composition, membrane choice, and electrode design toward more energy-efficient and scalable electrochemical processes.

## Introduction

Electrolysis enables the electrochemical breaking of chemical bonds to provide added-value reactions using renewable electricity. As energy usage is the primary driver of operating costs, we would like to minimize energy input, which by extension means trying to minimize the cell voltage that is driving the electrochemical reactions.^1,2^ Ideally, we would like to keep the cell voltage as close to the thermodynamic minimum as possible, while achieving high current densities.^3,4^

In electrochemical systems there are several sources contributing to cell voltage. These can be differentiated into the thermodynamic potential (*E*_eq_), which is the equilibrium potential to drive the electrochemical reactions at the electrodes, and the overpotentials (*η*), which are added on top of the thermodynamic contribution and account for extra energy barriers of the system. Sources of overpotentials can be ohmic losses due to ion or electron conduction (*η*_Ohm_), non-ohmic losses such as electrode kinetics (*η*_kin_), and mass transport-induced effects (*η*_conc_).^5–7^

The thermodynamic potential is the voltage equivalent of Gibbs free energy difference between products and reactants often expressed under standard conditions (*E*^0^). However, most electrolyzer initial conditions are not standard, and thus require a Nernstian correction (*e.g.* to our initial bulk concentrations). While the thermodynamic contribution sets the ideal reversible cell voltage, overpotentials play an important role in minimizing cell voltage. Under certain operating conditions, concentration polarization can lead to substantial changes in the local reaction environment, and lead to concentration overpotentials on the order of magnitude of the equilibrium potential.^8,9^

The most sizeable concentration overpotentials are pH-related shifts due to reaction-driven changes in proton (H^+^) and hydroxide (OH^−^) concentrations, which are present in most aqueous-based electrochemical reactions, such as water electrolysis, CO_2_ electrolysis, electrochemical H_2_O_2_ production and electrochemical ammonia production. In those cases, both Nernstian and concentration overpotentials are often expressed as a function of the pH (see Note S1). When a reaction has unity proton–electron stoichiometry at 25 °C the resulting overpotential is equivalent to 59 mV pH^−1^ unit penalty per electrode. As the current-dependent consumption or generation of H^+^ or OH^−^ occurs during electrochemical reactions, the pH at the electrode's surface shifts relative to the electrode's equilibrium condition (Fig. 1a). More precisely, in electrolysis the cathode experiences a pH increase due to H^+^ consumption or OH^−^ generation, while the anode undergoes a decrease in pH as a result of OH^−^ consumption or H^+^ generation. In other words, the system naturally evolves into local asymmetric pH conditions between both electrodes. The voltage effects of asymmetric pH conditions can be visualized on a Pourbaix diagram as shifting the cathode and anode potentials away from their equilibrium condition, leading to concentration overpotentials (Fig. 1b). The magnitude of these shifts depends heavily on the applied current density (Fig. 1c) and whether the initial pH conditions are poorly buffered (at near-neutral conditions) or more extreme pH conditions (*e.g.* pH < 1 or pH >13) (Fig. 1d). While high pH gradients are ideally avoided, fundamental chemistry or economics drives many electrochemical reactions into pH polarization.

**Fig. 1:**
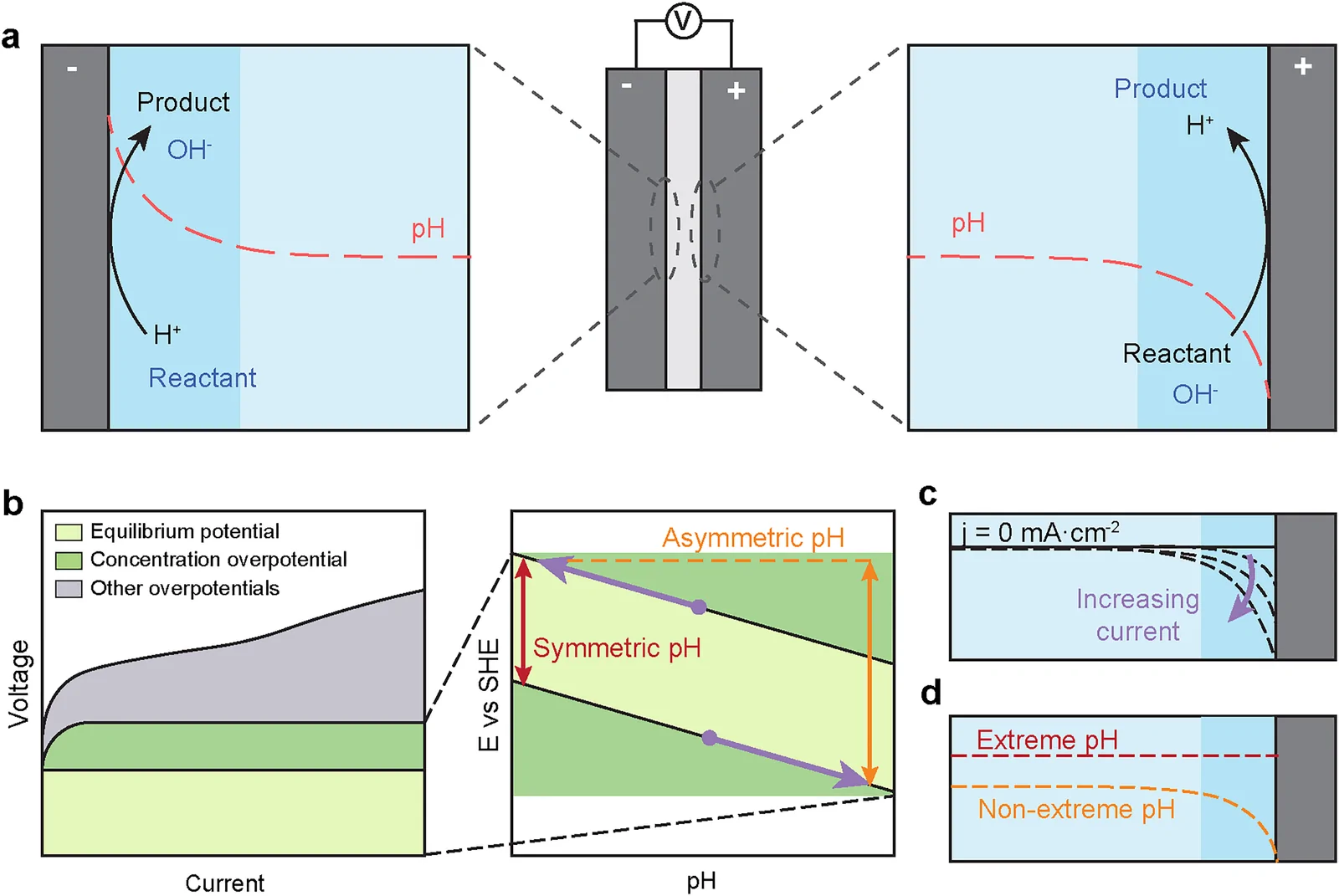
Transport-induced pH-asymmetry voltage penalty of membrane-electrode-assembly (MEA) electrolyzers operated under non-extreme pH conditions. (a) Concentration polarization develops local pH gradients at the electrodes surface from the consumption and production of H^+^ and OH^−^ in the electrochemical reactions resulting in local asymmetric pH cell conditions. The darker blue area represents the concentration boundary layer (local effects), while the lighter blue represents the bulk. (b) Cell voltage breakdown in equilibrium potential representing any initial state at equilibrium (*e.g.*: standard conditions or at different bulk pHs), concentration overpotential and other overpotentials (ohmic and kinetic). The pH asymmetry between cathode and anode reactions leads to a voltage penalty that can be represented in a Pourbaix diagram. The concentration overpotential arises from local pH gradients once a current is applied (purple arrows). (c) Increasing current density leads to larger pH shifts. (d) Extreme pH conditions can sustain the pH gradients and maintain symmetry, while non-extreme pH cases are susceptible to polarization leading to asymmetry between electrodes.

High concentration overpotentials pose a challenge for a group of non-water electrolysis reactions (*e.g.* CO_2_ reduction or nitrate reduction), which are driven inherently to non-extreme pH anolytes or catholytes by the feedstock source, p*K*_a_ of the reactants/products, and the ever-present competition with hydrogen or oxygen evolution at extreme pH conditions. In particular, the desire to bridge the gap between laboratory research and industrial applications will require many reactions to occur at current densities >100's mA cm^−2^, meaning that local proton and hydroxide consumption/generation will be substantial.^10,11^ Furthermore, the use of membrane-electrode-assembly (MEA) configurations typically inhibit liquid mass transport through micro/nanoporous catalyst layers, preventing the use of high fluid convection to reduce pH gradients. Concentration overpotentials then become more severe, leading to larger voltage penalties compared to operating at extreme pH conditions. Since novel reactions often follow a similar development pathway (from H-cell and catalysts studies to more industrially relevant cell configurations and operating conditions) these voltage penalties can be inherently carried through each stage. Awareness about these effects at earlier development stages would benefit us as a field. With electrons being a large cost driver of electrochemical processes,^12–14^ large pH asymmetry alone can present an insurmountable barrier.

There are many reasons to strive for non-extreme pH electrochemical reactions, such as lower costs of balance of plant, corrosion issues, lower shunt currents or to stabilize products and catalysts.^15,16^ We then want to generalize more clearly when asymmetric pH conditions will occur for the vast array of reactions taking place in aqueous media, and what is the expected penalty under high current density operation. It is then possible to see how pH symmetry can either be maintained or mitigated through operating conditions or external inputs.

In this work we demonstrate how and why electrochemical reactions with non-extreme pH in MEA cells lead to a transport-induced voltage penalty due to asymmetry. First, we use the simplest case of a water electrolyzer to show the correlation between the cell voltage and the initial pH conditions. Elaborating on this knowledge, we show how and why the cell voltages of semi-mature electrochemical reactions, such as CO_2_ or NO_3_^−^ reduction, are limited to much higher operational potentials than their standard potentials. Lastly, we extend this knowledge and provide specific scenarios on how we could avoid substantial concentration overpotentials in the presented cases and their possible drawbacks when applied in an industrially relevant scenario.

### Demonstrating pH asymmetry penalties in membrane electrode assembly water electrolysis

To show the relationship between cell voltage and pH asymmetry, we use the simple case of a water electrolyzer, with the hydrogen evolution reaction (HER) at the cathode and the oxygen evolution reaction (OER) at the anode. We combined several MEA configurations, with extreme pH and near-neutral pH electrolytes, that lead to various symmetric and asymmetric pH conditions at the electrodes as a function of current density (for detailed experimental conditions, see Note S2).

We first begin with the common cases in state-of-the-art water electrolyzers,^17^ where extremely acidic (PEM) or alkaline (AWE, AEMWE) electrolytes are used (Fig. 2a). Here the main charge carrier across the membrane or separator is either H^+^ or OH^−^, and is the same species generated at one electrode and consumed at the other. Due to the large abundance of H^+^ or OH^−^ present, the logarithmic change in local species is negligible. The highly acidic or alkaline conditions then maintain greater pH symmetry between electrodes (Fig. 2b). Upon running polarization curves for these conditions, we then show cell voltages <2 V (Fig. 2c) at elevated current densities, with lower current densities just above the standard cell potential of 1.23 V. For higher currents up to 200 mA cm^−2^, and for ohmic-corrected curves see Fig. S1 and S2.

**Fig. 2:**
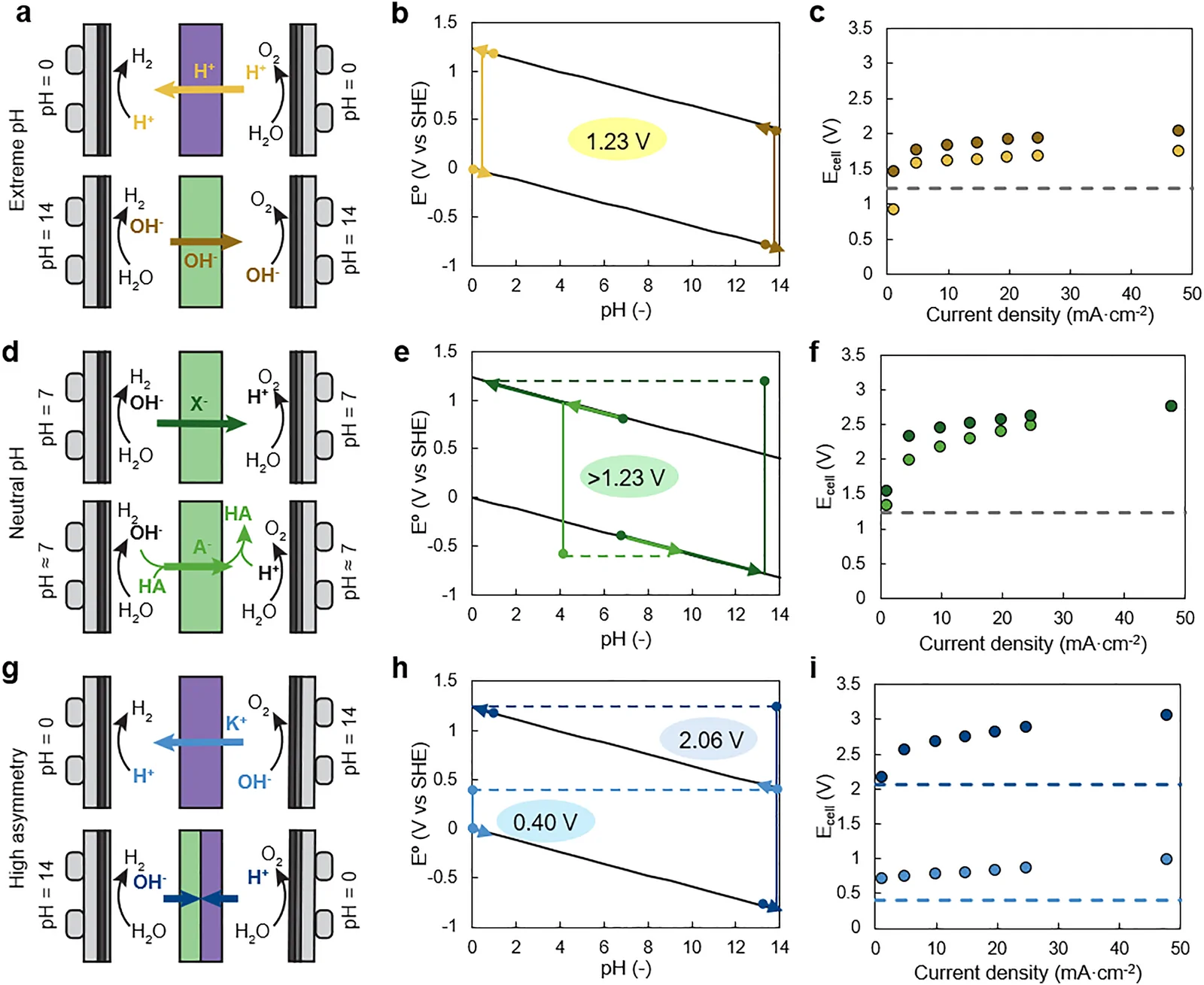
Cell voltages in membrane electrode assembly (MEA) water electrolyzers operated at extreme and non-extreme pH. (a, d and g). Scheme of a MEA water electrolyzer in which the cathode and the anode are fed with a liquid electrolyte of specific pH. (a) Extreme symmetric pH scenario, acidic (0.5 M H_2_SO_4_) and alkaline (1 M KOH) electrolytes. (d) Near-neutral pH with unbuffered (0.5 M K_2_SO_4_) and buffered conditions (1 M KHCO_3_). (g) Bulk asymmetric pH conditions, ΔpH = −14, with a catholyte of 0.5 M H_2_SO_4_ and an anolyte of 1 M KOH (and viceversa for ΔpH = 14). (b, e and h). Operational cell voltages predicted according to initial bulk pHs, Nernst shift and local concentration polarization effects. Under extreme pH (b) not much difference is expected from the equilibrium potential (1.23 V) while for near-neutral pH (e) the non-negligible polarization will cause large local pH shifts (>1.23). Under asymmetric pH conditions of ΔpH = −14 and ΔpH = 14 the equilibrium potential according to Nernst correction is expected to be 0.40 V and 2.06 V respectively. (c, f and i) Experimental polarization curves of the represented systems.

Under near-neutral pH conditions (Fig. 2d–f), however, both H^+^ and OH^−^ concentrations in the electrolytes are in the order of 10^−7^ M. The main charge carrier then changes to neutral species such as the present supporting anions (*e.g.*: SO_4_^2−^) in AEMs or alkali cations when using CEMs (Fig. 2d). Due to the low concentration of H^+^ and OH^−^, as current density increases water-splitting replaces the ions as a reactant, leading to OH^−^ and H^+^ by-products instead. This leads to local concentrations several orders of magnitude greater than the bulk concentrations.^18,19^ So when starting with symmetric near-neutral pH conditions, water electrolyzers will naturally evolve into local asymmetric systems deviating significantly from the equilibrium potential of 1.23 V as concentration overpotentials become significant (Fig. 2e). Experimentally, at only 5 mA cm^−2^ we observe cell voltages around 2.3 V increasing to 2.8 V at 50 mA cm^−2^, which corresponds to more than 1 V larger cell potentials *versus* the extreme pH cases (Fig. 2c). This behavior has been observed in seawater electrolysis and photochemical water splitting in which near-neutral conditions were employed.^20–23^

We also show the impact of buffers to help mitigate pH gradients by locally providing H^+^ or OH^−^ (Fig. 2d).^16,24^ However, their buffering capacity is limited and insufficient to maintain the local pH when applying higher current densities. Similar voltages for the unbuffered and buffered near-neutral pH electrolytes are then seen >50 mA cm^−2^ (Fig. 2f). Even further buffer engineering could allow for local pH buffering at higher current densities (≥100 mA cm^−2^).^25–27^ As cell voltage primarily affects operating expenditures, and current density more relates to capital expenditures, the use of buffers to limit cell potentials at lower current densities leads to a trade-off in operating *versus* capital expenditures that is application specific.

Notably the local pH under buffered conditions will depend on its acidity constant, often maintaining near-neutral pH conditions, which can lead to larger kinetic overpotentials.^23,28,29^ In addition, near-neutral pH electrolytes intrinsically have lower ionic conductivity than extreme pH, leading to a higher ohmic contribution (Fig. S3). However, we note that the ohmic drop is not the main reason for the observed difference between extreme and near-neutral pH conditions as it only accounts for 0–50 mV at the current densities of 1–50 mA cm^−2^. These trends are also maintained when exchanging the main charge carrier in near-neutral pH scenarios (SO_4_^2−^ or K^+^), by using a CEM instead of an AEM (Fig. S4). Similarly, the use of different catalysts and electrolytes for the OER and HER which have a direct effect on the kinetic overpotential cannot solely explain the observed differences (Fig. S5).

To further highlight the effects of local asymmetric pH, we added two extra cases that lead to unsustainable cell voltages (Fig. 2g). Here two scenarios are presented with initial bulk pH asymmetric conditions that have a ΔpH (cathode to anode) of −14 and 14 (Fig. 2g). Meaning the cathode is fed an acidic anolyte while the anode is fed an alkaline anolyte, and *vice versa* (see Note S2). We then expect the operational minimum potential to shift to 0.40 V and 2.06 V, respectively (Fig. 2h). Experimentally the trend holds, performing water electrolysis <1 V for the ΔpH = −14 case. Of course these voltages are not sustainable and are reliant on the external generation of H^+^ and OH^−^ which requires a minimum voltage of 0.83 V.^30^ On the contrary, in the ΔpH = 14 case, voltages are >2.5 V, reaching up to 3 V at 50 mA cm^−2^ (Fig. 2i). In some cases, this pH asymmetry is a requirement of the process that can still lead to a useful product, for example in the chlor-alkali process.^31^

Interestingly, when comparing the different polarization curves (Fig. 2c, f and i) it is seen that the near-neutral pH cases (Fig. 2f) approach the values of the artificial asymmetric case of a ΔpH = 14 (Fig. 2i). This infers that electrolyzers operating under near-neutral pH conditions naturally evolve into unfavorable asymmetric pH profiles once a current is applied.

While we did not perform any local pH measurement or characterize individual voltage contributions, here we infer that most of the voltage disparities observed come from concentration polarization effects. From the expected pH gradients generated we have estimated that concentration overpotentials can reach values up to 0.83 V (see Note S1) explaining mostly the large disparity in voltage. However, note that both HER and OER kinetics are dependent on catalyst selection and electrolyte conditions and might also contribute to the observed difference in voltage. In addition, in our assessment we have not included electrolyte Galvani-potential differences, such as Donnan potentials and membrane or liquid-junction potentials, which may add or compensate the electrode equilibrium potential shifts. In these cases, a proper analysis on the different ion electrochemical potentials and membrane transport numbers would be required. However, as we use symmetric electrolyte bulk conditions and the main membrane charge carrier is K^+^ or SO_4_^2−^ in near-neutral pH configurations (as compared to H^+^ nor OH^−^), the electrolyte-membrane voltage contributions are expected to be much smaller than the electrode equilibrium potential shifts.

### The reason why CO_2_ electrolyzers cannot operate <2 V when paired with OER

CO_2_ electrolysis enables the conversion of CO_2_ into value-added carbon products, such as carbon monoxide, ethylene, ethanol or methanol. Most often the cathodic CO_2_ reduction is paired with the Oxygen Evolution Reaction (OER) at the anode (Fig. 3a). Once these reactions are coupled together, we expect an equilibrium potential similar to water electrolysis (1.23 V for WE, 1.33 V for CO_2_ to CO, 1.15 V for CO_2_ to C_2_H_4_). Yet, state-of-the-art studies report cell voltages well above the 2 V range (Fig. 3b). Several studies have aimed to breakdown voltage distributions within CO_2_ electrolyzers,^5,32^ while others link high voltages to shifts in the equilibrium cell potential,^33,34^ local effects such as Nernstian shifts^35^ or concentration overpotentials,^36^ or other overpotentials. However, these findings have largely been discussed independently, and there has not been a unifying framework that underlines the origin of the higher observed cell potentials. Inspired by these works, here we propose a detailed explanation on why and how CO_2_ electrolyzers operate under locally asymmetric pH conditions, leading to a ∼0.5 V penalty in the cell voltage. And notably, the conditions which would be required to break this asymmetry.

**Fig. 3:**
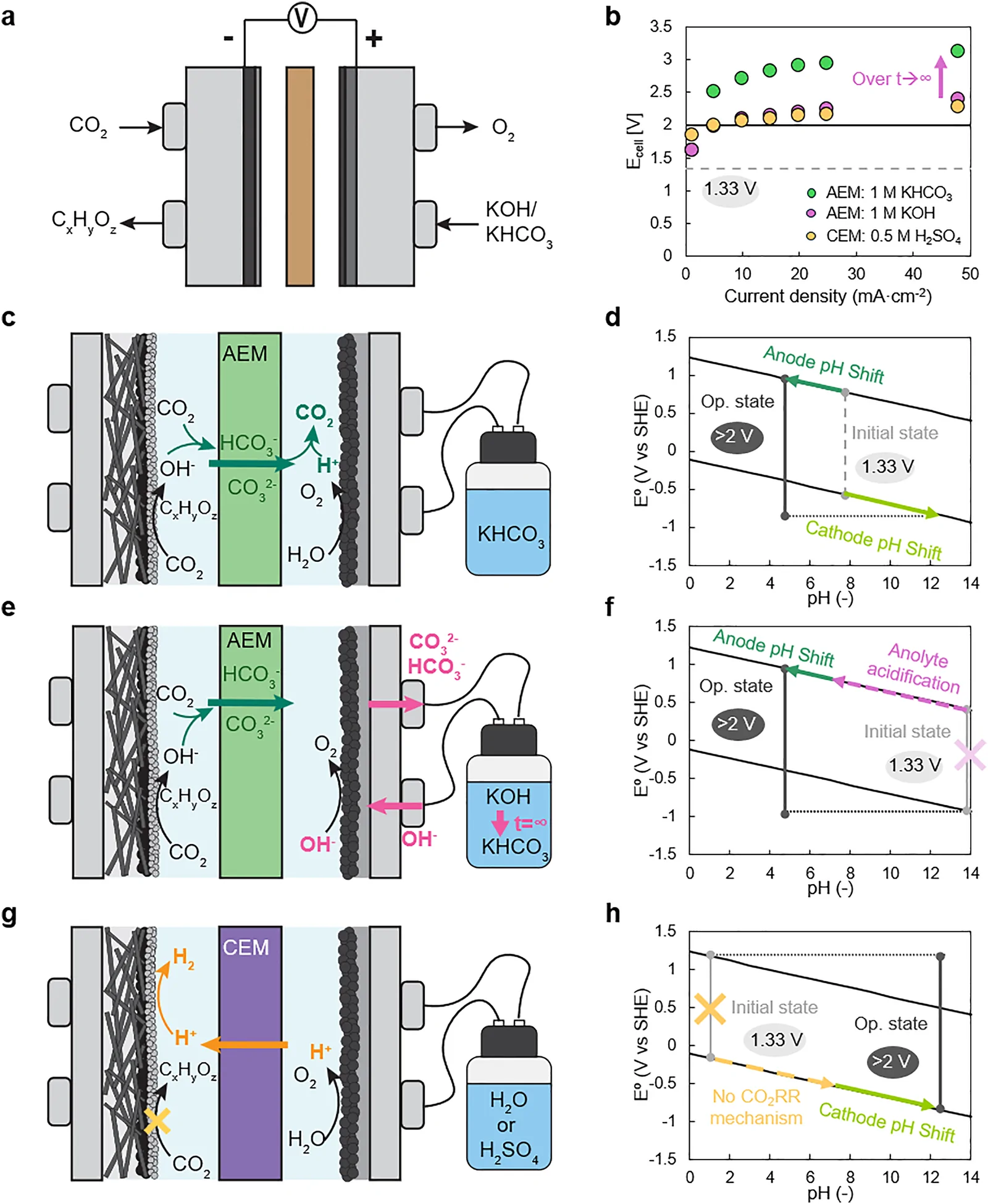
Cell voltages penalties and their origin in CO_2_ electrolysis. (a) Scheme of a MEA CO_2_ electrolyzer. (b) Experimental polarization curves for MEA CO_2_-to-CO electrolyzers (CO_2_ + H_2_O + 2e^−^ → CO + 2OH^−^). Cases (membrane + anolyte) AEM + 1 M KHCO_3_, AEM +1 M KOH and CEM + 0.5 M H_2_SO_4_ (which led to FE(CO) = 0%, see Fig. S9). (c) Crossover of (bi)carbonate species from cathode to anode when using an AEM. CO_2_ evolution at the anode from H^+^ formation. (d) (Bi)carbonate formation and crossover in AEM CO_2_ electrolyzers shift the operational minimum voltage from 1.33 V to 2 V. (e) The consumption of OH^−^ by the OER and (bi)carbonate crossover causes acidification of the anolyte converting KOH into KHCO_3_ over time. (f) The use of KOH as anolyte initially allows to minimize the anodic concentration overpotential, but carbonate crossover and acidification shifts again the bulk anolyte towards near-neutral pH conditions (Nernstian shift). (g) CO_2_ electrolysis under extremely local acidic conditions (pH ≤ 1) is still not existent and even then will face selectivity issues due to competition with HER. (h) As CO_2_ reduction initialization requires a pH shift from acidic conditions to near-neutral pH, the system inherently leads to a pH ∼ 7 and further local alkalinization. Straight arrows represent polarization pH shifts (concentration overpotential), while dotted arrows represent pH shifts caused by non-local phenomena (Nernstian shifts).

The main origin of the pH asymmetry in a CO_2_ electrolyzer is the formation of (bi)carbonate species from acid–base reactions between the CO_2_ supplied to the cathode and the OH^−^ generated during CO_2_ reduction (eqn (1) and (2)). In the CO_2_ reduction mechanism, water acts as the proton donor, causing hydroxide as a by-product. However, this hydroxide reacts with CO_2_, causing the local generation of (bi)carbonate shifting the cathode local pH towards alkaline conditions.^37–40^ Consequently, the concentration of (free) hydroxides is then too low to be used for oxygen evolution at the anode, causing water oxidation at the anode to produce H^+^. When using an AEM, the carbonate formed then crosses to the anode as the main charge carrier and reacts with H^+^ from the OER (eqn (3)) causing the anolyte to remain at a bicarbonate-buffered near-neutral pH (eqn (4))^41–46^ (Fig. 3c). While carbonate is a buffer, its buffering capability is insufficient at higher current densities leading to non-negligible local pH shifts at the anode and cathode.^11,39,47^ Therefore, the natural alkalinization and acidification of both cathode and anode, respectively, results in asymmetric pH conditions and thus a voltage penalty shifting the operational minimum voltage to around 2 V (Fig. 3d). Practically, when accounting for kinetic and ohmic overpotentials, the cell voltage reaches 2.5V–3.0 V, which is 2-fold higher than the standard cell potential (Fig. 3b, for full polarization curves up to 200 mA cm^−2^ or iR corrected curves, see Fig. S6).

1 CO_2_RR: CO_2_ + H_2_O + 2e^−^ → CO + 2OH^−^

2 CO_2_ + 2OH^−^ ↔ HCO_3_^−^ + OH^−^ ↔ CO_3_^2−^ + H_2_O

3 OER: 2H_2_O → O_2_ + 4H^+^ + 4e^−^

4 CO_3_^2−^ + 2H^+^ ↔ HCO_3_^−^ + H^+^ ↔ CO_2_ + H_2_O

More symmetric pH conditions in CO_2_ electrolysis can be artificially maintained when operating with an alkaline anolyte (*e.g.* KOH). Here the concentration polarization at the anode is mostly mitigated, while the cathode region is driven to high alkalinity by the combination of CO_3_^2−^ and OH^−^. Thus, by using 1 M KOH anolytes we can decrease the overall cell voltage by almost 1 V as both concentration and kinetic overpotential are favorable under alkaline conditions (Fig. 3b). While maintaining symmetry is initially possible, the anolyte will be converted back to near-neutral pH conditions due to the continuous crossover of carbonate species through the AEM, and consumption of OH^−^ by the OER (Fig. 3e). The local anodic pH will then shift again towards acidic conditions leading to an asymmetric pH cell profile, and thus to the minimum observable cell voltage of 2 V (Fig. 3f). While this strategy is still used in the field by using larger anolyte volumes or regenerating the anolyte after certain number of hours, and indeed can lead to artificial lower cell voltages, its application on an industrial scale is unfeasible.^48^ The same problem arises when a cation exchange membrane (CEM) combined with an alkaline anolyte (*e.g.* KOH) is used (see Fig. S7).

The use of acidic conditions offers another way of enabling symmetric pH conditions by pushing the local (bi)carbonate equilibrium back to CO_2_.^49^ However, to achieve locally symmetric acidic conditions, we need the use of a CEM which transports the H^+^ generated by the OER from the anode to cathode, similar to a PEMWE (Fig. 3g). The biggest issue of such configuration is that so far, CO_2_ reduction in locally acidic conditions has not yet been demonstrated.^50,51^ A recent study^52^ shows that CO_2_RR occurs only once a certain pH swing at the electrode takes place. They show that a minimum pH around 6–7 is required for CO_2_ to be reduced. So while large concentration polarization at the anode may be avoided, the cathode is likely to experience a full shift from locally acidic to locally alkaline, again leading to a minimum cell voltage of >2 V (see orange and green lines in Fig. 3h).

There are several works using mildly acidic conditions (pH > 1) that benefit from the presence of H^+^ to avoid carbonate crossover and CO_2_ losses.^53–56^ However, their local pH is shown to be alkaline, and thus the voltage penalty is still there. Even if CO_2_R could be performed under local acidic conditions, it would still need to kinetically compete with the Hydrogen Evolution Reaction (Fig. S8).^57^

Other strategies that can minimize carbonate crossover, such the use of bipolar membranes^44,58–60^ still suffer from the voltage penalty as they inherently lead to asymmetric pH conditions. Under forward-bias (f-BPM),^61^ since the AEL faces an alkaline cathode and the CEL an acidic anode, it naturally will operate under unfavorable asymmetric pH conditions and thus will have near-unity concentration overpotentials (∼0.7 V with dpH = 12) leading to a minimum operational voltage >2 V (Fig. S9). On the contrary, in reverse-bias (r-BPM) water molecules are dissociated into H^+^ and OH^−^, which are driven towards the cathode (CEL) and anode (AEL), respectively, at a cost of 0.83 V.^62^ However, r-BPM could only operate close to the equilibrium cell potential of 1.33 V (0.5 V from electrode reactions and 0.83 V from water dissociation) if the cathode and anode are fully operated at extreme acidic and alkaline pH, respectively. However, due to the necessary alkalinity required for CO_2_ reduction and the presence of carbonate crossover which shifts the anodic operation towards acidic conditions,^44,48^ the minimum operational cell voltage in r-BPM CO_2_ electrolyzers approaches the 3 V range (Fig. S10).

Therefore, currently avoiding asymmetric pH conditions in CO_2_ electrolyzers seems unfeasible when paired with the OER, both due to mechanistic prohibition and industrial application limitations, requiring at least 2 V to carry the electrochemical reactions. Alternative anodic reactions such as glycerol oxidation or ethylene/ethanol oxidation may decrease the overall cell voltage by replacing the OER with lower potential reactions.^63–65^ However, since the reactions take place in aqueous media where H^+^ and OH^−^ are involved as reactants or products, the inherent problems described here will also be occurring (*e.g.*: carbonate crossover), and thus the voltage penalty be present.

### Near-neutral pH electrolyzers and their voltage penalty

In the experimental results from the previous sections, we have shown the transport-induced voltage penalties of aqueous-based electrolyzers when operated under near-neutral pH conditions. Here, we would like to extend the more well-understood cases to one where asymmetrical pH implications have yet to be considered. This includes any electrochemical reaction that due to its nature occurs under near-neutral pH and includes H^+^ or OH^−^ within the reaction mechanism, such as seawater electrolysis,^66^ nitrate reduction, urea synthesis,^67^ biomass electroreduction^68^ or other alternative oxidation reactions (AOR).^69^ In this section we apply our discussion to NO_*x*_ electrolyzers.

Nitrate reduction offers a pathway towards producing NH_3_ or by remediating wastewater by converting it to N_2_.^70,71^ Even though the feasibility of NH_3_ reduction as a replacement to Haber–Bosch process is questionable,^72^ its potential has led to many studies in this field. Most often the nitrate reduction reaction is paired with the oxygen evolution reaction, and its combination has been widely implemented in a MEA configuration (Fig. 4a).^73,74^

**Fig. 4:**
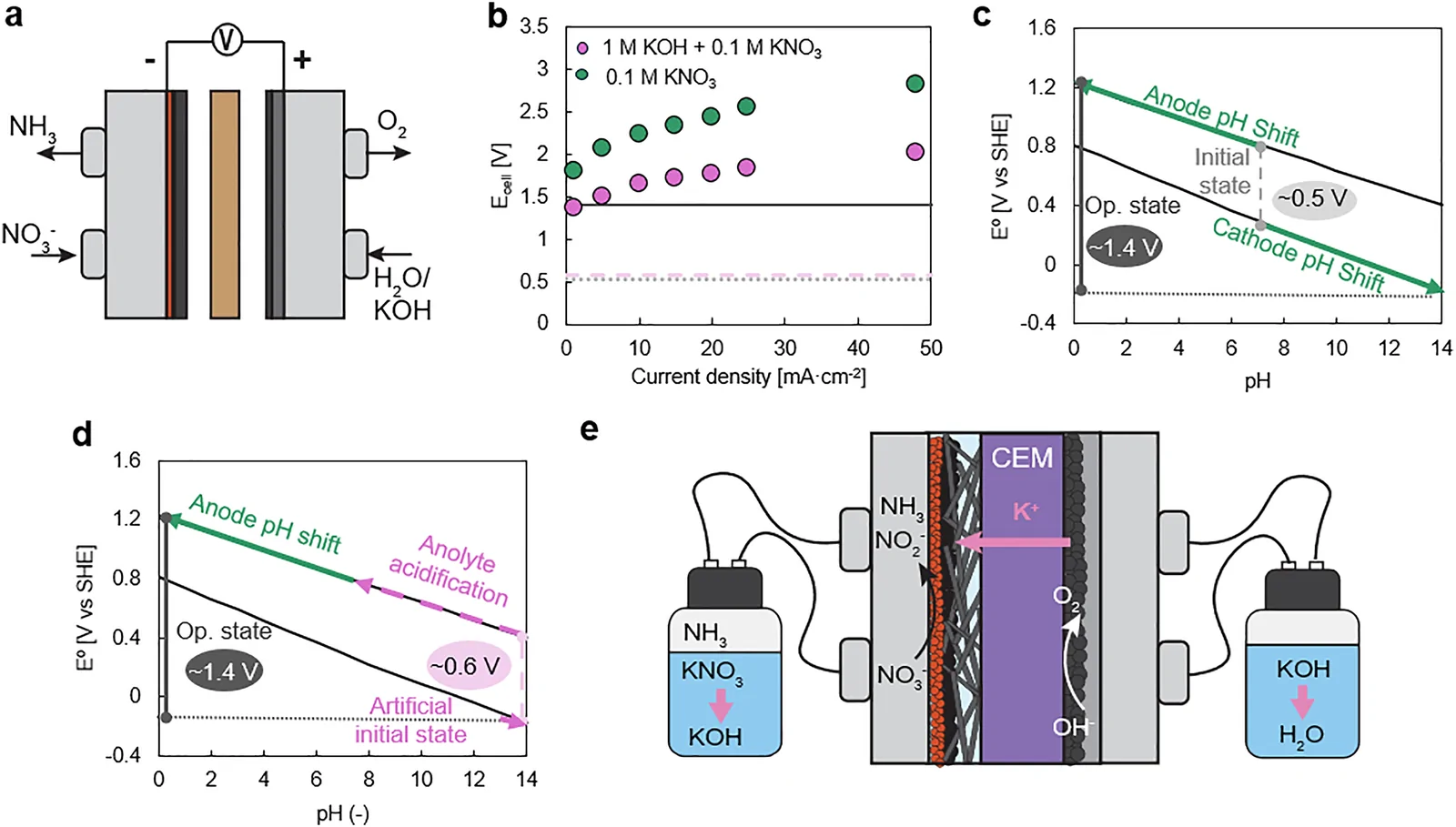
Nitrate reduction cell voltages dependance on electrolyte conditions. (a) NO_*x*_ electrolyzer MEA cell configuration. Nitrate reduction to ammonia (NO_3_^−^ + 6H_2_O + 8e^−^ → NH_3_ + 9OH^−^) at the cathode and the oxygen evolution reaction at the anode. (b) Polarization curves with alkaline (adding 1 M KOH both catholyte and anolyte) and near-neutral bulk pH conditions (0.1 M KNO_3_). (c) Under near-neutral pH conditions the initial state (0.5 V) cannot be sustained as local pH gradients lead to a higher operational minimum cell potential (1.4 V) (d) with the addition of an alkaline supporting electrolyte, nitrate electrolyzers can maintain pH conditions and minimize local pH gradients. However, over time the net consumption of OH^−^ at the anode will lead to near-neutral pH conditions and thus asymmetry will appear. (e) Over time both catholyte and anolyte bulk conditions will be transformed into asymmetric pH conditions, as the transport across the membrane does not enable the replenishment of OH^−^ at the anode. Straight arrows represent polarization pH shifts (concentration overpotential), while dotted arrows represent pH shifts caused by non-local phenomena (Nernstian shifts).

Nitrate feedstock usually comes from nitrate-rich waste streams such as industrial wastewater or polluted groundwater from agriculture applications. Even after concentrating such streams, its pH remains at near-neutral pH conditions. As such we performed nitrate reduction experiments in an MEA cell in a similar manner to the water and CO_2_ electrolysis experiments, to examine asymmetry under different electrolyte conditions. Similar to the neutral pH water electrolysis experiments without buffering, the cell potential for a 0.1 M KNO_3_ stream exceeds 1.8 V at just 5 mA cm^−2^ (Fig. 4b), much higher than the difference in standard redox potentials of 0.5 V (Fig. 4c). Note that nitrate species do not act as buffers, thus the pH gradients will be observable at even lower current densities.^75,76^ For the full polarization curves and faradaic efficiencies including the cathode nitrogen balance see Fig. S11 and S12, respectively.

The use of extreme pH conditions in nitrate electrolyzers has also been widely achieved by adding an alkaline supporting electrolyte to both the nitrate feedstock catholyte and the anolyte (*e.g.* 1 M KOH). While such alkalization avoids some near-neutral pH disadvantages such as poor kinetics and lower selectivity towards N-products,^77–79^ another indirect benefit is the minimization of Nernstian shifts as shown in Fig. 4d. Upon performing experiments with KOH, the observed cell voltage indeed drops substantially *versus* the 0.1 M KNO_3_ case (Fig. 4b).

Notably in the above nitrate experiments a CEM is commonly used, which is similar to other works. Here K^+^ acts as the charge carrier towards to cathode while OH^−^ is consumed at the anode. The case of nitrate reduction then falls into a similar, but different pitfall, compared to CO_2_ electrolysis. Here hydroxide within the anolyte is consumed by OER but hydroxide is not replenished through ionic transport due to the use of a CEM. Depletion over time then leads to bulk pH asymmetry (Fig. 4e). Meanwhile the KNO_3_ catholyte is converted into NH_3_ and KOH, effectively causing a net transfer of hydroxide from the anolyte to the catholyte. While NH_3_ generated at the cathode can be recovered in the gas phase, the overall pH balance of the system requires the cathode-generated KOH to be fully recycled to the anolyte. Such an anolyte recycling process is more complicated than mixing the catholyte and anolyte, as the source of nitrate is not a recirculating electrolyte volume, but rather a continuous low-concentration environmental feedstock. Not only is the expected KOH concentration then more dilute, but a recovery process needs to be implemented which will incur their own energy costs. So, while alkaline solutions are not acidified as in the case of CO_2_, operating under extreme pH leads to system stability issues.^80^

The underlying source of the problem in this case is the CEM which forces K^+^ transport. Changing to an anion exchange membrane, could also allow for extreme pH conditions to be used (Fig. S13), however the unwanted crossover of anionic species such as NO_3_^−^ or NO_2_^−^ must also be prevented to avoid slow hydroxide depletion.^81^ As discussed in our other work for reverse-bias bipolar membranes for CO_2_ electrolysis, even a 1% co-ion crossover can lead to rapid pH destabilization in a few hundred hours.^48^

While we have not provided experiments for other electrochemical reactions with a non-zero proton–electron stoichiometry, we expect similar trends and behaviors to restrict other systems from operating under extreme pH conditions, either due to selectivity or stability issues. When operation at near-neutral pH is required, the pH asymmetry voltage penalty will then be a challenge to overcome.

### Outlook: overcoming asymmetrical pH penalties

In previous sections we have shown how evolving asymmetric pH conditions cause a voltage penalty in near-neutral pH electrolyzers, with an emphasis on MEA cells where control of local conditions is more challenging. Here, we approach it from a different perspective. Based on our observed criteria for pH asymmetry to arise in paired electrochemical reactions we ask: which solutions can we implement to prevent local pH gradients and what challenges could arise from them, especially when applied in an industrial scenario? As pH asymmetry develops when the rate of H^+^ or OH^−^ generation/consumption at the electrodes exceeds the rate at which these ions are transported in/out the membrane or bulk electrolyte (Fig. 5a), we expect that by designing strategies that can artificially minimize the pH gradients developed we could overcome the voltage penalties. In general, these strategies aim to decrease the concentration gradients formed within a catalyst layer through a mixture of transport and chemical methods.

**Fig. 5:**
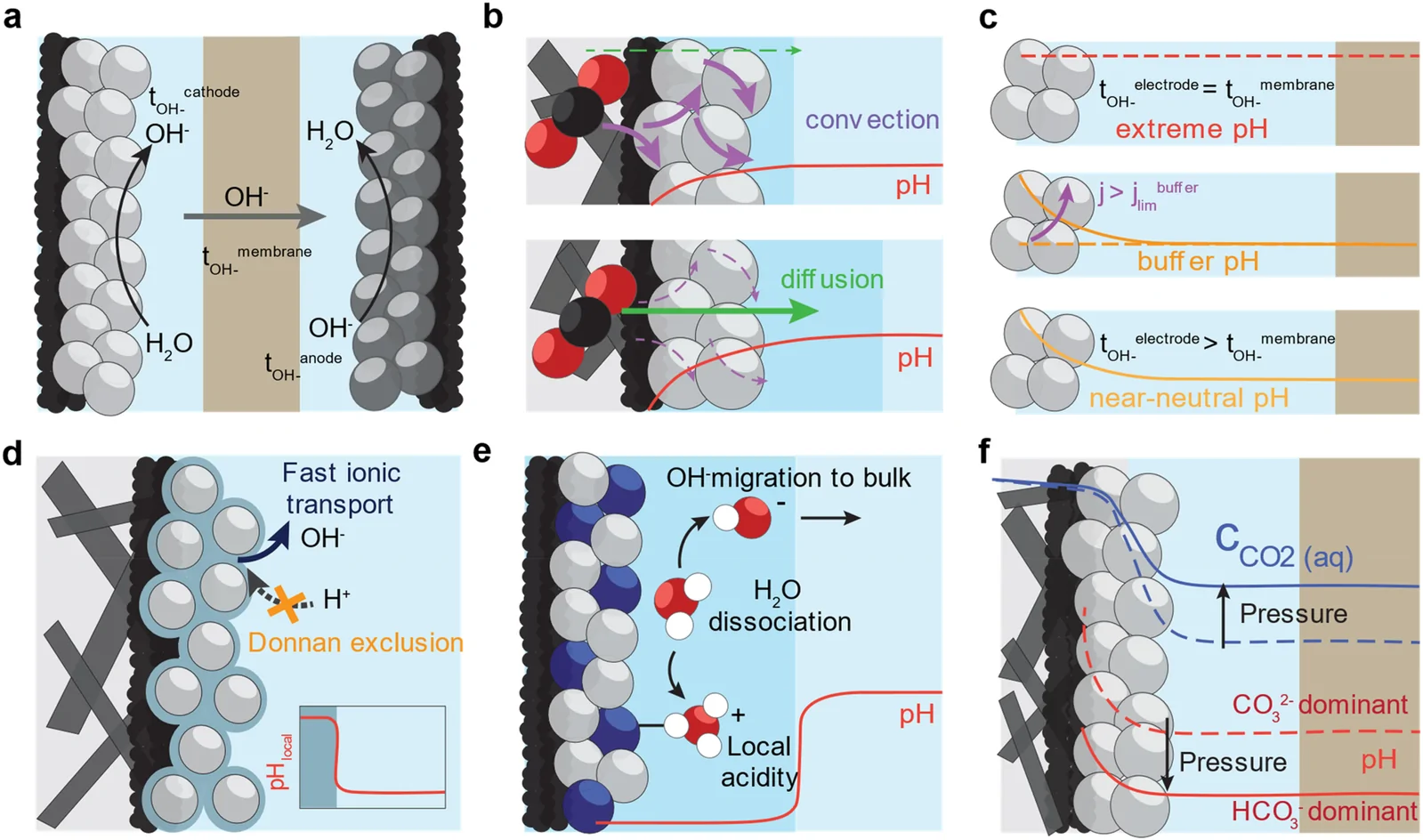
Strategies to minimize local pH shifts that lead to non-negligible Nernstian losses. (a) Local pH gradients arise when local OH^−^ generation or consumption at the electrodes cannot be sustained by ionic transport through the membrane (*t*_OH^−^_ represents OH^−^ transport number). (b) Convection enhances mass transport of reactants and products decreasing the boundary layer. A smaller boundary layer (darker blue) minimizes local pH shifts. Bulk represented in lighter blue. (c) The use of supporting electrolytes modifies bulk composition which tunes the local environment at the electrolytes through ion transport. (d and e). Modulating local chemical environment with the use of ionomers and inorganic redox electrodes. (d) Example of an anionic ionomer around the catalyst that enables fast ionic transport (OH^−^) and excludes oppositely charged (H +) *via* Donnan exclusion. (e) Example of an inorganic redox material dissociating H_2_O molecules to create a locally acidic environment. The H^+^ generated are attracted by the negative field of the cathode, while the OH^−^ migrate to the anode. (f) Pressure increases CO_2_ solubility which changes buffering chemistry of (bi)carbonate. Higher pressures result in milder pH shifts as more CO_2_ can neutralize the OH^−^ produce and maintain the buffer at bicarbonate conditions.

One of the simplest means of decreasing concentration gradients within the catalytic region is to increase mass transport to the reaction surface. Conceptually we can think of increasing mass transport as also decreasing the concentration boundary layer where diffusive effects are dominant (Fig. 5b). The thickness and structure of the concentration boundary layer can then be tuned by the hydrodynamics at the electrodes. The simplest version of this is increasing the fluid pumping rate through a cell. However, within an MEA configuration the catalyst is embedded behind the micro/nanoporous transport layers which inhibits high velocities near the catalyst layer. Changes to configurations or electrolyte feed strategies are then required to meaningfully reduce concentration overpotentials. In CO_2_ reduction, we have seen many examples in which mass transport has been increased to maximize the availability of CO_2_ at the reaction sites. Most notably Wen *et al.* showed a decrease of 10-fold in the boundary layer thickness by changing the typical flow-by configuration of conventional GDEs to a system where CO_2_ is delivered in a dissolved form that this forced directly through the catalyst layer.^82^ Such a scenario represents the lowest non-KOH cell voltage reported in literature at higher current densities, reaching 100 mA cm^−2^ at just above 2 V. In their work they show that not only is CO_2_-availability boosted, but also that the local pH gradients are minimized. Other means of reducing concentration gradients include the use of different end plate flow fields^83^ (*e.g.* interdigitated flow fields) which force electrolyte into the porous transport layers, although such approaches are limited in effect and are only applicable to liquid phases. For gas-fed CO_2_ electrolyzers only the anode pH gradient can then be fixed for example. A final mass transport-oriented approach are works that replace traditional GDEs with foam replacements. For example a recent work has shown that 3D foam fields can increase convective mass transport of reactants leading to 200–300 mV voltage decrease respect conventional parallel and serpentine flow fields.^84^ The use of higher temperatures can also enhance the mass transport in/out the electrodes as diffusion and migration are both boosted, thus reducing the diffusive boundary layer. However, the net effect of the temperature on the local pH temperature is more complex than that as temperature also affects electrode kinetics, buffering or CO_2_ solubility, which can significantly impact the local pH.^85,86^

As shown in previous sections, buffering reactions or supporting extreme pH electrolyte can also be used to moderate the consumption or generation of H^+^ and OH^−^ near the electrode surface.^15,87^ Here the supporting electrolyte is able to counter a formed pH gradient, up until the point where current densities are so high that the buffer breaks. Buffers are then applicable only up to certain current density regimes, with their buffering capacity depending on concentration and kinetics^88^ (Fig. 5c).

An alternative approach to modify the local chemical environment at the electrodes is by engineering the catalyst interface with the use of ionomers^89^ or inorganic redox electrodes.^90,91^ Ionomers can provide ion-conducting pathways for faster transport of H^+^ and OH^−^ or encapsulate locally H^+^ and OH^−^*via* Donnan exclusion, both helping to regulate local pH^92–96^ (Fig. 5d). Supporting materials such as metal oxides catalysts or hydrous transition metal oxides, can act as H^+^ sources by dissociating locally water molecules and creating local acid-like microenvironments, or encapsulating OH^−^ to increase the local pH^97–101^ (Fig. 5e). Similarly, in CO_2_ electrolyzers the use of pressure can locally scavenge OH^−^,^102^ by increasing the abundance of CO_2_ which buffers the local environment and minimizes the pH shifts (Fig. 5f).

## Conclusions

Our work shows that any aqueous electrochemical reaction, with H^+^ or OH^−^ consumption and generation, and operating under non-extreme pH conditions, will have large pH gradients at the electrodes. Here we demonstrate that a transport-induced voltage penalty is observed in water, CO_2_ and NO_*x*_ electrolyzers when asymmetric pH conditions develop between the electrodes. These pH shifts, caused by a combination of reaction-specific phenomena and fundamental ion transport in the cell, can incur concentration overpotentials on the order of magnitude of the thermodynamic equilibrium potential (0.5–1 V). As electrochemical technologies move towards industrial applications, and electricity costs remain one of the major drivers, decreasing the concentration overpotentials arisen from operating in non-extreme pH conditions is a requirement for industrialization. Consequently, we propose strategies to minimize local pH gradients, such as decreasing the concentration boundary layer by enhancing ionic transport to the electrodes, the use of buffers, or by modulating the electrode local environment with the use of ionomers or redox inorganic species. Identifying and quantifying the pH gradients generated in our cell in earlier development stages, as well as research on strategies that can mitigate them, thus becomes essential to achieve and maximize the energy efficiency and economic viability of our electrolyzers.

## Experimental

### Electrochemical testing

All electrolysis experiments (water, CO_2_ and nitrate) were performed in a 5 cm^2^ membrane electrode assembly cell (MEA) from Dioxide Materials. The cell was composed of a cathode and anode (see Electrodes preparation in methods for more details), separated with a membrane, all compressed at a torque of 2.5 N m.

In water electrolysis, both the cathode and the anode were liquid-fed with an electrolyte. The electrolyte was pumped and recirculated at 20 mL min^−1^ (Fig. S14). For each configuration shown a combination of different electrolytes were used (1 M KOH, 1 M KHCO_3_, 0.5 M H_2_SO_4_ or 0.5 M K_2_SO_4_). The main charge carrier in the cell was adjusted by choosing the appropriate membrane (anion-exchange membrane (AEM) – Piperion 40 µm, cation-exchange membrane (CEM) – Nafion 115, or bipolar membrane (BPM) – Fumasep FBM). For specific details on the cell composition for the different experiments, check Note S2.

In CO_2_ electrolysis the cathode was fed with 50 sccm of humidified CO_2_. The cathode outlet volumetric flow was determined by the MFM and its products quantified by a gas chromatograph (GAS Compact 4.0, see Note S5 for calculations). The anolyte, composed of 1 M KOH or 1 M KHCO_3_, was continuously recirculated at 20 mL min^−1^ (Fig. S15). The membrane was exchanged depending on the experiment between an AEM (Piperion 40 µm), CEM (Nafion 115) or BPM (Fumasep FBM). For detailed experimental conditions for all experiments, see Note S3.

In nitrate electrolysis, the cathode was facing with the catalyst layer towards the flow channels instead of the membrane.^73^ The catholyte used was a solution of 0.1 M KNO_3_ with or without addition of 1 M KOH. It was fed in a single-pass configuration, in which the outlet was captured in an acid trap (160 mL of 0.5 M H_2_SO_4_). From the acid trap, samples were taken to measure the quantity of NH_4_^+^, NO_3_^−^ and NO_2_^−^*via* ion chromatography (Metrohm 883 Basic ion chromatography with a Metrosep C6 column for NH_4_^+^, and a Metrosep supp 5 column for NO_3_^−^ and NO_2_^−^). From these concentrations the faradaic efficiencies and the nitrogen balance were calculated (see Note S6). The anolyte (1 M KOH under alkaline conditions, 1 M KNO_3_ under near-neutral pH), was constantly recirculated (Fig. S16). Both electrolytes were pumped at 5 mL min^−1^ for all current densities. For detailed experimental conditions, see Note S4.

### Polarization curves

The polarization curves were obtained by performing constant current steps (chronopotentiometry) using a potentiostat (Biologic). Steps at 1, 5, 10, 15, 20, 25, 50, 100, 150 and 200 mA cm^−2^ were performed, followed with galvanostatic EIS measurements at the specified current density (10^5^–1 Hz, 5 mA amplitude). The EIS measurements were used to correct for ohmic drop by obtaining the high frequency resistance (*R*_ohmic_) (Fig. S17). Each current was sustained for 5 minutes (Fig. S18). For CO_2_ and nitrate electrolysis experiments, in which the faradaic Efficiency was calculated the time was extended to 20 minutes for the currents in which selectivity was measured (10, 25, 50, 100, 200 mA cm^−2^).

### Electrodes preparation

Two different anodes were used depending on the pH experimental conditions. Under highly alkaline conditions (in 1 M KOH anolytes) a nickel foam (Ni-4753.005 from Recemat BV) was used. In acidic and near-neutral pH conditions an iridium coated titanium felt anode (0.6 mg cm^−2^ from HPNow) was used.

In water electrolysis, again depending on the pH we varied the catalyst. Under alkaline conditions we used the same nickel foam (Ni-4753.005 from Recemat BV), while for acidic or near-neutral pH conditions the cathode was prepared by sputtering 100 nm of Pt onto a Sigracet 39AA gas diffusion layer. Similarly, in CO_2_ and nitrate electrolysis, the cathodes were prepared by sputtering 100 nm of Ag onto a Sigracet 39BB gas diffusion layer and 300 nm of Cu onto a Sigracet 39AA, respectively.

## Author contributions

Conceptualization: G. P. V., T. B. Experimental methodology: G. P. V, Y. Z, M. L. Experimental data and data analysis: G. P. V, Y. Z, M. L. Visualization: G. P. V, T. B. Funding acquisition: T. B. Supervision: D. V, T. B. Writing – review & editing: G. P. V, D. V., T. B.

## Conflicts of interest

The authors declare no competing financial interest.

## Supplementary Material

EY-OLF-D6EY00240D-s001

## Data Availability

All data is made available in the manuscript and the supplementary information (SI). Supplementary information: detailed experimental conditions employed during water, CO_2_ and nitrate electrolysis experiments. Data showing full polarization curves, ohmic correction, faradaic efficiencies and extra schemes to explain specific phenomena. See DOI: https://doi.org/10.1039/d6ey00240d. Raw data made available through deposition on the 4TU.Centre. See DOI: https://doi.org/10.4121/6bd99d09-2370-4133-b89f-2cc542dc3e73.
