# Supplementary material for: Transport-induced voltage penalties of aqueous electrochemical reactions involving proton or hydroxide transfer in non-extreme pH conditions
Source: EES Catal. 2026 Sep 23. Online ahead of print. doi: 10.1039/d6ey00240d (PMC13628322; doi:10.1039/d6ey00240d)
Supplement: Supplementary file 1 [file EY-OLF-D6EY00240D-s001.pdf]

Supplementary Information (SI) for EES Catalysis.
This journal is © The Royal Society of Chemistry 2026

Transport-induced Voltage Penalties of Aqueous Electrochemical Reactions Involving Proton or Hydroxide Transfer in non-extreme pH conditions

Gerard Prats Vergel[1,2], Yvette Ziggers[1], Min Li[1], David A. Vermaas[1,2], Thomas Burdyny[1,2] *

*Corresponding author email: T.E.Burdyny@tudelft.nl

[1] Department of Chemical Engineering, Faculty of Applied Sciences, Delft University of Technology, van der Maasweg 9, 2629 HZ Delft, The Netherlands.

[2] e-Refinery Institute, Delft University of Technology, Leeghwaterstraat 39, 2628 CB Delft, The Netherlands

Table of Contents

Supplementary Notes....................3
Supplementary Note 1 – Equilibrium potential, Nernst and concentration overpotential....................3
Supplementary Note 2 – Water electrolysis experimental conditions....................6
Supplementary Note 3 – CO_2 electrolysis experimental conditions....................7
Supplementary Note 4 – Nitrate electrolysis experimental conditions....................7
Supplementary Note 5 – Faradaic Efficiencies for CO_2 electrolysis....................8
Supplementary Note 6 – Faradaic Efficiencies for nitrate electrolysis....................8
Supplementary Data....................10

Supplementary Notes

Supplementary Note 1 – Equilibrium potential, Nernst and concentration overpotential

In this work we have used several times the terms "equilibrium potential", "Nernst shifts" and "concentration overpotential". As this is sometimes confusing in the field, we would like to explicitly describe what we mean with each term in our work.

Here, we define the cell potential (E_{cell} in Equation 1) as a function of the equilibrium thermodynamic potential (E_{eq}), the kinetic overpotential (η_{kin}), the concentration overpotential (η_{conc}) and the ohmic contribution (iR) (Equation 1).

$$E_{cell} = E_{eq} + \eta_{kin} + \eta_{conc} + iR \tag{1}$$

The thermodynamic potential refers to the potential required to drive an electrochemical reaction assuming equilibrium (E_{eq}). This equilibrium state is often represented under standard conditions (standard redox potential E^0). However, most of our reactions do not occur under standard conditions, and thus a correction is required. In that case, the Nernst equation is used (Equation 2, for the following reaction: $aA + bB \rightarrow cC + dD$, where a_i represents the activity of specie "i"). Here, we uniquely refer to Nernstian shifts as thermodynamics shifts, basically a shift between initial states respect to standard conditions or specific bulk conditions. For example, when the anodic bulk conditions in a CO_2 electrolyzer shift from 1 M KOH to 1 M $KHCO_3$ due to carbonate crossover, or when in the water electrolysis cases we use artificial asymmetric bulk pH conditions to shift the minimum voltage observed.

$$E_{eq} = E^o - \frac{RT}{zF} lnQ_{bulk} = E^o - \frac{RT}{zF} \ln \left(\frac{a_C^c \cdot a_D^d}{a_A^a \cdot a_B^b} \right)_{bulk} \tag{2}$$

Current-dependent potential differences fall in the overpotentials domain, as they shift away from the equilibrium state that defines the equilibrium potential. Thus, anything that pushes the local conditions out of the equilibrium is considered overpotential. Specifically, processes related to mass transfer, such as local pH gradients, are included inside the concentration overpotentials (Equation 3). The concentration overpotential thus arises from differences in concentrations between bulk and surface. For example, in our work this represents the local pH shifts in non-extreme pH conditions caused by concentration polarization.

$$\eta_{conc} = E_{bulk} - E_{surface} = \frac{RT}{zF} ln\left(\frac{c_{bulk}}{c_{surface}}\right) \tag{3}$$

Note that Equation 2 and Equation 3 have a similar shape, but here we consider their origin to be different. The concentration overpotential accounts for local current-induced differences in concentrations (so surface – bulk differences), while the Nernstian equation aims to correct for non-standard conditions at a specific initial state (no current applied, different initial activities than standard conditions).

These equations are generally expressed as function of activities (Equation 4 and 5, for gases and in solutions respectively) but are often simplified to concentrations. For very dilute conditions (1mM) $\gamma \sim 0.95 - 1$, for moderate concentrations (0.01-0.05 M) $\gamma \sim 0.8 - 0.9$, and for concentrated (0.5 – 1 M) $\gamma \sim 0.4 - 0.6$.

$$a_i = \frac{f_i}{p^o} = \varphi_i \cdot y_i \cdot \frac{p}{p^o} = \varphi_i \cdot \frac{p_i}{p^o} \tag{4}$$

$$a_i = \gamma_i \cdot \frac{c_i}{c^o} \tag{5}$$

Here, we want to illustrate how both Nernstian shifts and concentration overpotentials can be represented as a function of pH. To do that, we use the case of CO_2 reduction to CO (Equation 6) and the initial Nernst equation (Equation 2). A similar process can be applied for the concentration overpotential if instead of bulk conditions, operating local conditions are considered. In CO_2 reduction the Nernst equation can be expressed as Equation 7.

$$CO_2 + H_2O + 2e^- \rightarrow CO + 2OH^- \tag{6}$$

$$E = E^o - 2.303\frac{RT}{2F}\log_{10}\frac{a_{OH^-}^{2} \cdot a_{CO}}{a_{CO_2} \cdot a_{H_2O}} \tag{7}$$

First, we can set, $a_{H_2O} \sim 1$ and separate the activity coefficients term from the concentration and partial pressures to study the effect of each correction term (Equation 8).

$$E = E^o - 2.303\frac{RT}{2F}\log_{10}\frac{\left(\gamma_{OH^-} \cdot c_{OH^-}\right)^2 \cdot \left(\varphi_{CO} \cdot p_{CO}\right)}{\left(\varphi_{CO_2} \cdot p_{CO2}\right)} =$$

$$= E^o - 2.303\frac{RT}{2F}(\log_{10}(\frac{\left(\gamma_{OH^-}\right)^2 \cdot \varphi_{CO}}{\varphi_{CO_2}}) + \log_{10}\left(\frac{\left(c_{OH^-}\right)^2 \cdot p_{CO}}{p_{CO2}}\right)) =$$

$$= E^o - 2.303\frac{RT}{2F}(\log_{10}(\frac{(\gamma_{OH^-})^2 \cdot \varphi_{CO}}{\varphi_{CO_2}}) + \log_{10}\left(\frac{p_{CO}}{p_{CO2}}\right) + \log_{10}\left((c_{OH^-})^2\right)) \quad (8)$$

The fugacity coefficients of gases like CO_2 and CO are approximately ~ 1 at the pressures we operate our CO_2 electrolyzers (1-10 bar). Even then, a fugacity coefficient of 0.9 would only result in ~1 mV difference. In addition, when considering a coefficient activity of 0.4 for OH^- (for concentrated solutions), we see that its term approximates to a few decades of mV (~-20mV) Thus, the impact of fugacity and activity coefficients is often negligible.

The ratio of CO and CO_2 depends on the composition of the gas mixture and can vary due to several reasons such as spatial effects, gas flow rate or current density. At low conversions (90% CO2, 10% CO) the contribution would have a positive shift of 30mV. Oppositely, at high conversion (10% CO2, 90% CO), it will have a 30 mV penalty.

Finally, when converting the OH^- concentration into H^+, an equation as a function of pH can be obtained (Equation 9). At 25°C, the Nernstian losses equal to ~ 59 mV per pH unit. At higher temperatures, the slope increases (e.g: 80ºC is ~70 mV per pH unit). Since, pH swings in electrochemical reactions can reach several units due to local polarization, the pH-related voltage losses can go up to several 100s of mV. Note that, even though pH seems to be the most significant factor to potential deviations, it is still very important to study each own system and see how the effect of each of these variables such as pressure, temperature or concentration of reactants and products might deviate our cell voltage from the standard values.

$$E \approx E^o - 2.303\frac{RT}{F}pH \quad (9)$$

So, according to Equation 9, operating an electrolyzer under asymmetric pH conditions with a ΔpH = 14, can result in a voltage penalty of 0.83 V at 25ºC, but it can increase up to 0.98 V at 80ºC. Similarly, for milder pH asymmetry such as ΔpH = 8 -10 (more common in CO_2 electrolyzers), the voltage penalty related to concentration overpotential would be of 0.47 – 0.59 V at 25ºC.

Supplementary Note 2 – Water electrolysis experimental conditions

To simulate the pH dependent water electrolyzers configurations shown in Fig. 2 of the manuscript we have used the experimental conditions shown in Supplementary Table 1.

Table 1. Experimental conditions for the MEA water electrolysis experiments.

Experiment	Cathode	Catholyte	Anode	Anolyte	Membrane*
Extreme pH – acidic	Sigracet 39AA – Pt 100 nm	0.5 M H_2SO_4	Ir coated titanium felt (0.6 mg·cm^{-2} from HPNow)	0.5 M H_2SO_4	Nafion 115
Extreme pH - alkaline	Ni foam	1 M KOH	Ni foam (Ni-4753.005 from Racemat BV)	1 M KOH	Piperion 40μm
Near-neutral pH – unbuffered	Sigracet 39AA – Pt 100 nm	0.5 M K_2SO_4	Ir coated titanium felt (0.6 mg·cm^{-2} from HPNow)	0.5 M K_2SO_4	Nafion 115
Near-neutral pH unbuffered	Ni foam (Ni-4753.005 from Racemat BV)	0.5 M K_2SO_4	Ir coated titanium felt (0.6 mg·cm^{-2} from HPNow)	0.5 M K_2SO_4	Piperion 40μm
Near-neutral pH – buffered	Sigracet 39AA – Pt 100 nm	1 M $KHCO_3$	Ir coated titanium felt (0.6 mg·cm^{-2} from HPNow)	1 M $KHCO_3$	Nafion 115
Near-neutral pH buffered	Ni foam (Ni-4753.005 from Racemat BV)	1 M $KHCO_3$	Ir coated titanium felt (0.6 mg·cm^{-2} from HPNow)	1 M $KHCO_3$	Piperion 40μm
Asymmetric pH – dpH = -14	Sigracet 39AA – Pt 100 nm	0.5 M H_2SO_4	Ni foam (Ni-4753.005 from Racemat BV)	1 M KOH	Nafion 115
Asymmetric pH – dpH = 14	Ni foam (Ni-4753.005 from Racemat BV)	1 M KOH	Ir coated titanium felt (0.6 mg·cm^{-2} from HPNow)	0.5 M H_2SO_4	Fumasep FBM-PK operated under forward-bias

* Nafion 115 membranes have been stored in DI or 1 M KOH. Piperion 40 μm in 1 M KOH. Fumasep FBM-PK in 1% wt. KCl.

Supplementary Note 3 – CO_2 electrolysis experimental conditions

To simulate the CO_2 electrolyzers configurations shown in Fig. 3 of the manuscript we have used the experimental conditions shown in Supplementary Table 2.

Table 2. Experimental conditions for the MEA CO_2 electrolysis experiments.

**Experiment**	**Cathode**	**Anode**	**Anolyte**	**Membrane***
State-of-the-art AEM $KHCO_3$	100 nm of Ag sputtered on Sigracet 39 BB	Ir coated titanium felt (0.6 mg·cm^{-2} from HPNow)	1 M $KHCO_3$	Piperion 40µm
Extreme pH - alkaline		Ni foam (Ni-4753.005 from Racemat BV)	1 M KOH	Piperion 40µm
Extreme pH - acidic		Ir coated titanium felt (0.6 mg·cm^{-2} from HPNow)	0.5 M H_2SO_4	Nafion 115
Reverse-bias BPM		Ni foam (Ni-4753.005 from Racemat BV)	1 M KOH	Fumasep FBM
Forward-bias BPM		Ir coated titanium felt (0.6 mg·cm^{-2} from HPNow)	0.5 M H_2SO_4	Nafion 115

* Nafion 115 membranes have been stored in DI or 1 M KOH. Piperion 40 µm in 1 M KOH. Fumasep FBM-PK in 1% wt. KCl.

Supplementary Note 4 – Nitrate electrolysis experimental conditions

To simulate the NO_x electrolyzers configurations shown in Fig. 4 of the manuscript we have used the experimental conditions shown in Supplementary Table 3.

Table 3. Experimental conditions for the MEA nitrate electrolysis experiments.

**Experiment**	**Cathode**	**Catholyte**	**Anode**	**Anolyte**	**Membrane**
Alkalinized electrolyte	300 nm of Cu sputtered on Sigracet 39 AA	0.1 M KNO_3 + 1 M KOH	Ni foam	1 M KOH	Nafion 115
Near-neutral pH		0.1 M KNO_3	IrOx	1 M KNO_3	Nafion 115

Supplementary Note 5 – Faradaic Efficiencies for CO_2 electrolysis

The Faradaic efficiencies for the gas products were calculated using Eq. 10:

$$FE_{i,gas} = \frac{z \cdot P \cdot Q_{gas} \cdot c_i \cdot F}{R \cdot T \cdot I} \tag{10}$$

Where P is the pressure (101325 Pa), Q_{gas} is the volumetric flow rate of the gases and c_i the concentration of product i measured by the GC. F corresponding to the Faraday constant (96485 $C \cdot mol^{-1}$), R is the gas constant (8.3145 $m^3 \cdot Pa \cdot K^{-1} \cdot$mol-1, T the temperature, z the number of charges transferred in the electrochemical reaction, and I the total current.

The volumetric flow rate of the gases, Q_{gas}, is measured by a mass flow meter calibrated for CO_2. To convert the measured CO_2-calibrated flowrate from to flowrate (Q_{gas}) we use the following equations:

$$Q_{gas} = \frac{Q_{MFM}}{C_{CO_2}} C_{mix} \tag{11}$$

$$C_{mix} = \frac{1}{\sum_i \frac{c_i}{C_i}} \tag{12}$$

Q_{MFM} is obtained from the last 30 seconds flowrates registered by the MFM before a GC injection. C_{mix} is the gas mixture conversion factor obtained from the volume fraction of the gases (c_i) and their gas conversion factor (C_i), being 0.74 for CO_2, 1.00 for CO, 1.01 for H_2 and 0.79 for H_2O vapor. It is assumed 100% relative humidity.

Supplementary Note 6 – Faradaic Efficiencies for nitrate electrolysis

The Faradaic Efficiencies in nitrate electrolysis were obtained from samples extracted from the catholyte. The ions i measured were nitrate (NO_3^-), nitrite (NO_2^-) and ammonium (NH_4^+). These concentrations were obtained in ppm units. To convert to molarity Equation 13 was used. To account for the dilution factor from the acid trap (c_{total}), we used Equation 14 in which $V_{acid\ trap}$ was the volume of 0.5M H_2SO_4 used (160 ml) and V_{flowed} the volume flowed over the 20 minutes of operation.

$$c_i^{sample}(M) = c_i^{sample}(ppm) \cdot \frac{1}{1000 \cdot M_w} \tag{13}$$

$$c_i^{total} = c_i^{sample} \cdot \frac{V_{flowed} + V_{acid\ trap}}{V_{flowed}} \tag{14}$$

From the molarities a nitrogen balance can be performed, between the initial electrolyte and the post-electrolysis one, to check for mass conservation (Equation 15).

$$c^{initial}_{NO_3^-} = c^{post}_{NO_3^-} + c^{post}_{NO_2^-} + c^{post}_{NH_4^+} \tag{15}$$

In addition, the Faradaic efficiencies for each of the products (NH_4^+, NO_2^- and H_2) can be also obtained from the concentrations (Equation 16). In the equations, F corresponds to the Faraday constant (96485 $C \cdot mol^{-1}$), z the number of charges transferred in the electrochemical reaction, t the time of measurement (20 minutes), j the current density ($mA \cdot cm^{-2}$) and A the electrode area (cm^{-2}). The H_2 Faradaic efficiency (Equation 17) is derived assuming that the total Faradaic efficiency is 1, and subtracting the FEs for both NH_4^+ and NO_3^-.

$$FE_i = \frac{z \cdot F \cdot V_{flowed} \cdot c^{post}_{i}}{t \cdot j \cdot A} \tag{16}$$

$$FE_{H_2} = 1 - FE_{NH_4^+} - FE_{NO_2^-} \tag{17}$$

Supplementary Data

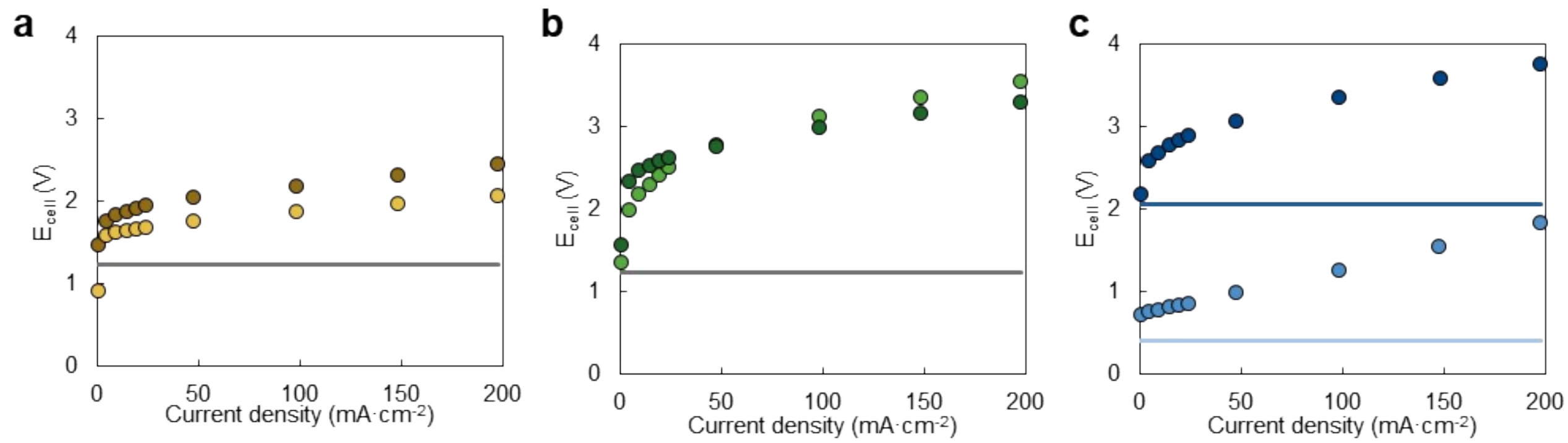

Fig. 1. Full polarization curves (1-200 mA·cm^{-2}) water electrolysis (Fig. 2 in Manuscript). a. Extreme pH scenarios (acidic 0.5 M H_2SO_4 and alkaline 1 M KOH). **b.** Near-neutral pH scenarios (unbuffered 0.5 M K_2SO_4 and buffered 1 M $KHCO_3$), and **c.** Asymmetric pH scenarios. ΔpH = -14, with a catholyte of 0.5 M H_2SO_4 and an anolyte of 1 M KOH (and viceversa for ΔpH = 14)

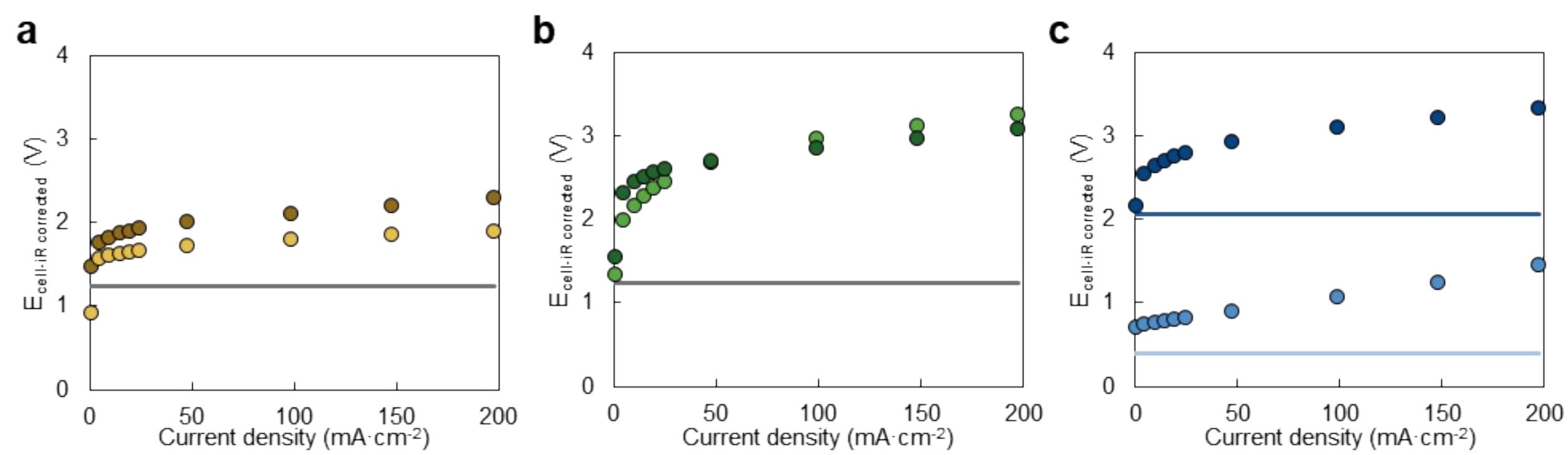

Fig. 2. Ohmic-corrected polarization curves (1-200 mA·cm^{-2}) water electrolysis (Fig. 2 in Manuscript). a. Extreme pH scenarios (acidic 0.5 M H_2SO_4 and alkaline 1 M KOH). **b.** Near-neutral pH scenarios (unbuffered 0.5 M K_2SO_4 and buffered 1 M $KHCO_3$), and **c.** Asymmetric pH scenarios. ΔpH = -14, with a catholyte of 0.5 M H_2SO_4 and an anolyte of 1 M KOH (and viceversa for ΔpH = 14)

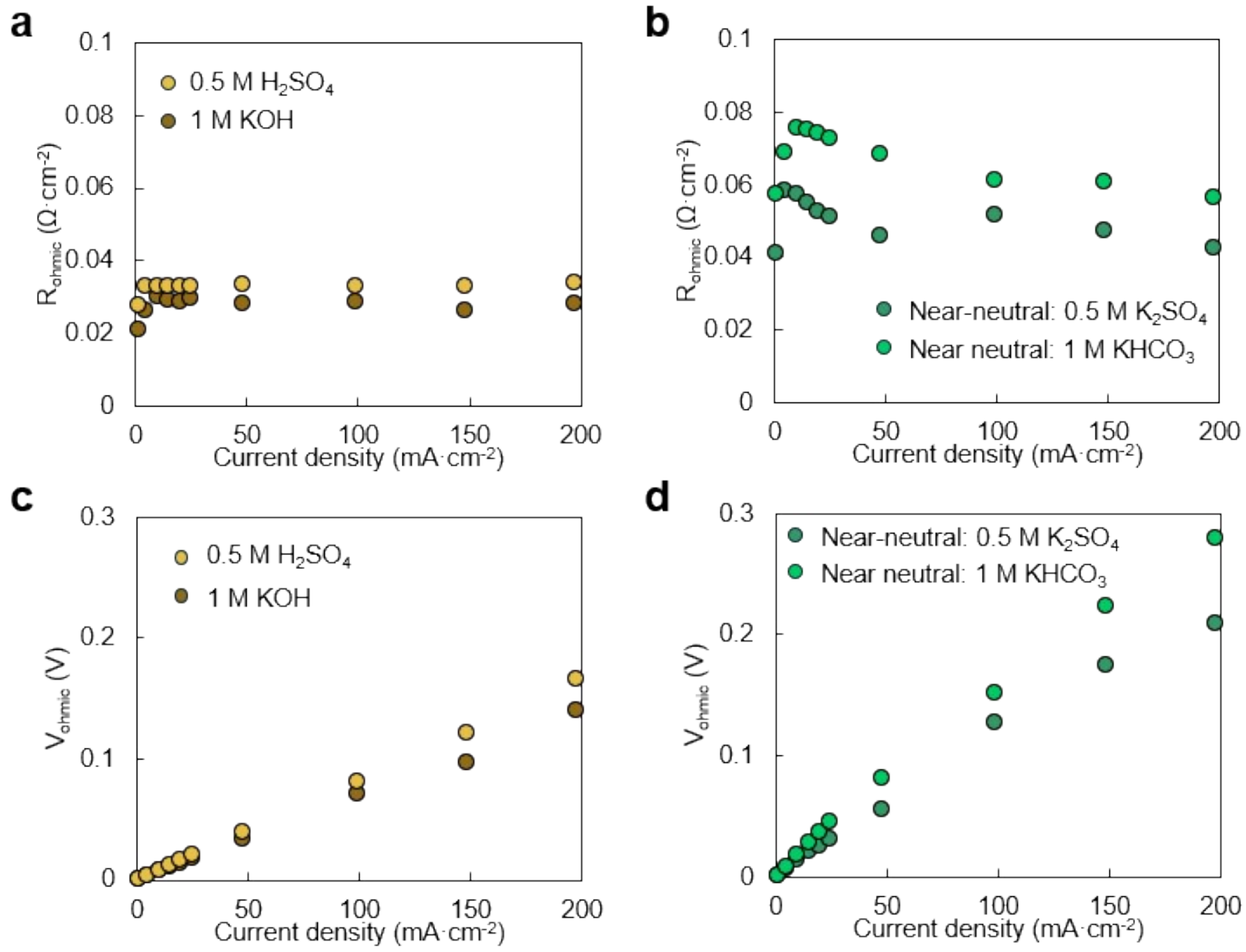

Fig. 3. Cell ohmic contribution for extreme pH (acidic 0.5 M H_2SO_4 and alkaline 1 M KOH) vs near-neutral pH (unbuffered 0.5 M K_2SO_4 and buffered 1 M $KHCO_3$) water electrolyzers. a, b. Ohmic resistance ($\Omega\cdot cm^{-2}$). **c, d.** Voltage penalty due to Ohmic resistance (V).

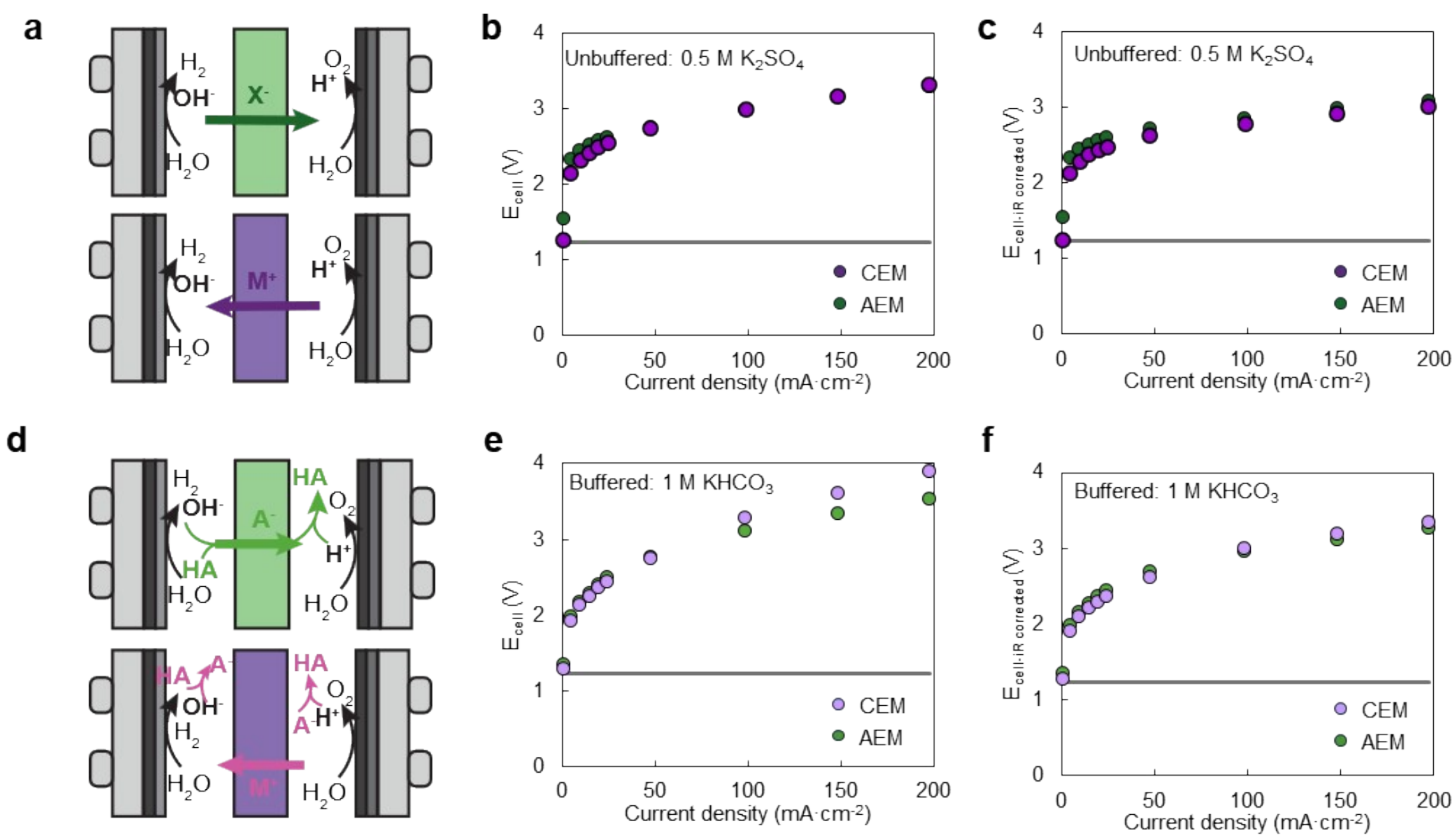

Fig. 4. Near-neutral pH water electrolysis. The use of AEM or CEM does not influence the polarization curves. **a, d.** Scheme of water electrolyzer with **a,** unbuffered (0.5 M K_2SO_4 **d,** buffered (1 M $KHCO_3$) conditions. **b, c, e, f.** Polarization curves (**c, f**, Ohmic-corrected). Green represents an AEM and purple a CEM.

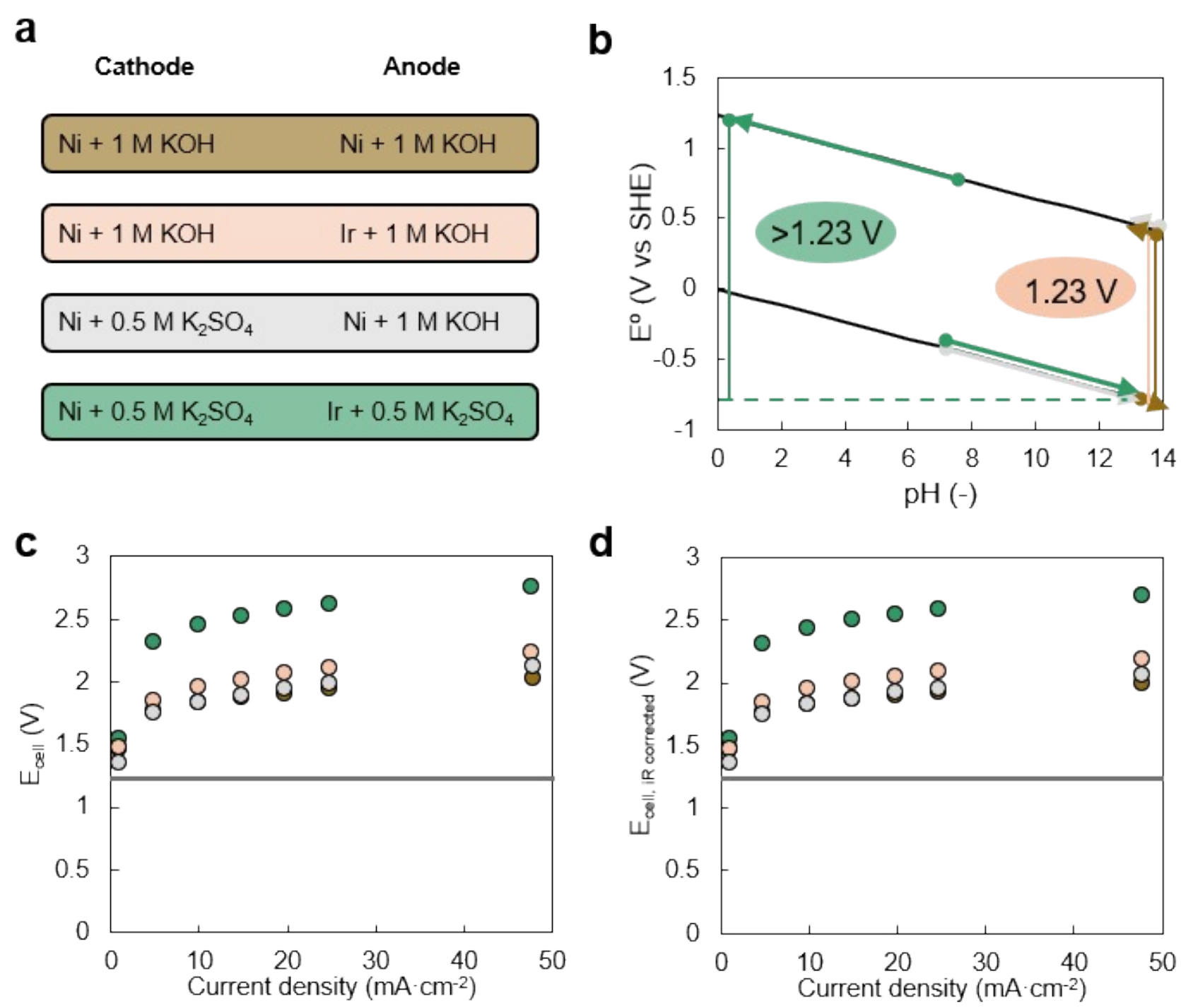

Fig. 5. Effects of catalyst and bulk electrolyte in measured cell voltage. a. Cathode and anode selection, with respective catholyte and anolyte for each of the experiments. In all experiments an AEM was used (Piperion 40 µm). Due to stability reasons, under near-neutral conditions Ni anodes were not used. **b.** Expected polarization overpotential for each of the cases represented. Only when operating both anode and cathode with near-neutral pH electrolyte leads to local asymmetric pH conditions, and a minimum operational cell voltage >1.23 due to large concentration overpotentials. **c,d.** The measured cell voltages show a few hundred mV difference when changing the catalyst and the electrolyte mostly due to kinetic effects and ohmic resistances. However, the selection of catalyst and electrolyte fail to fully explain the 0.8 V difference when using near-neutral pH conditions both at the anode and cathode, inferring the concentration overpotential can play a big role to the observed voltage discrepancies.

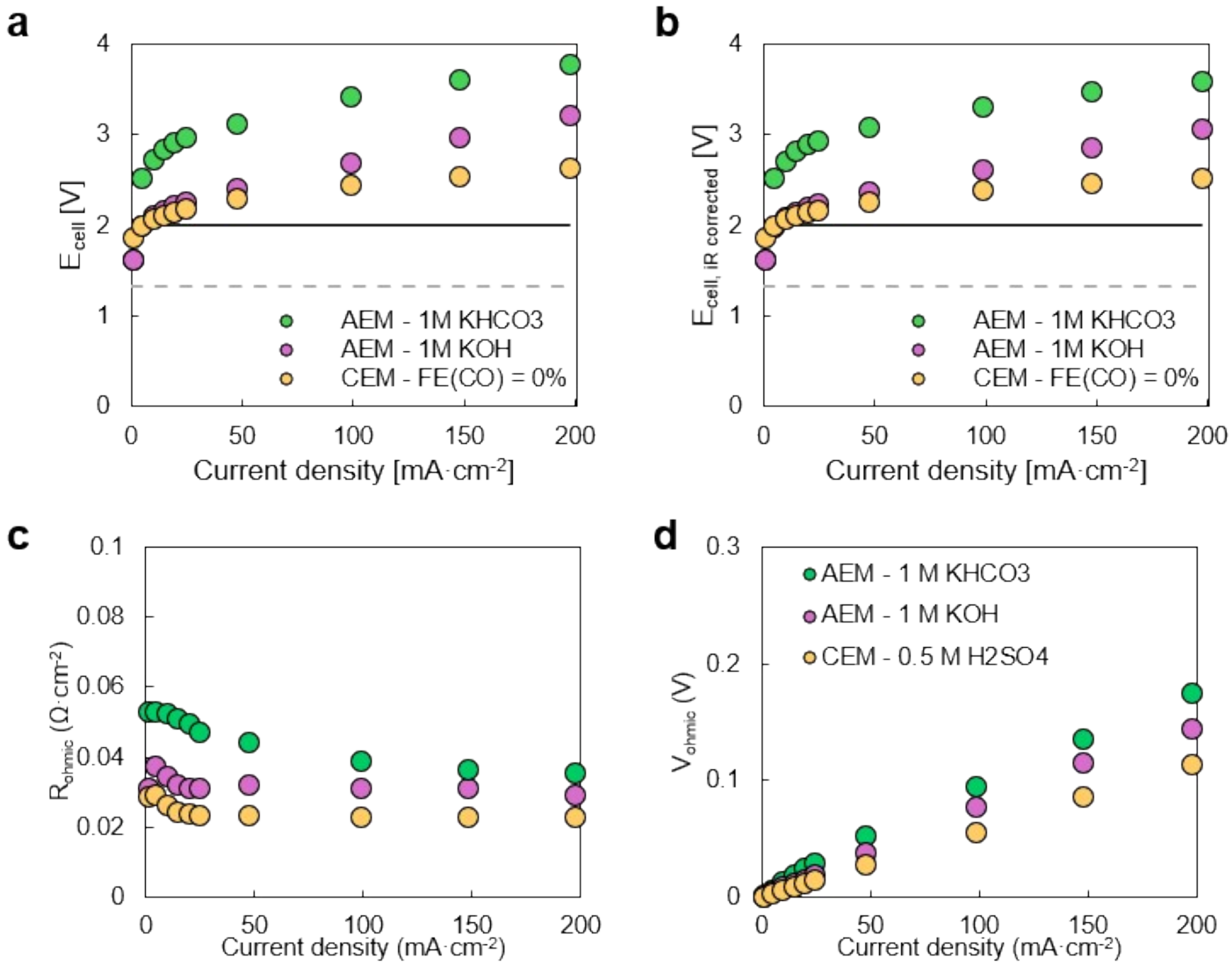

Fig. 6. Full polarization curves (1-200 mA·cm^{-2}) CO_2 electrolysis (Fig. 3 in Manuscript) and Ohmic contribution. Polarization curves **a,** non-corrected and **b,** corrected for Ohmic drop. **c,** Ohmic resistance (Ω·cm^{-2}). **d,** Voltage penalty due to Ohmic resistance (V). Cases represented include MEA with an AEM using a 1 M $KHCO_3$ and a 1 M KOH anolyte, and a CEM with a 0.5 M H_2SO_4 anolyte.

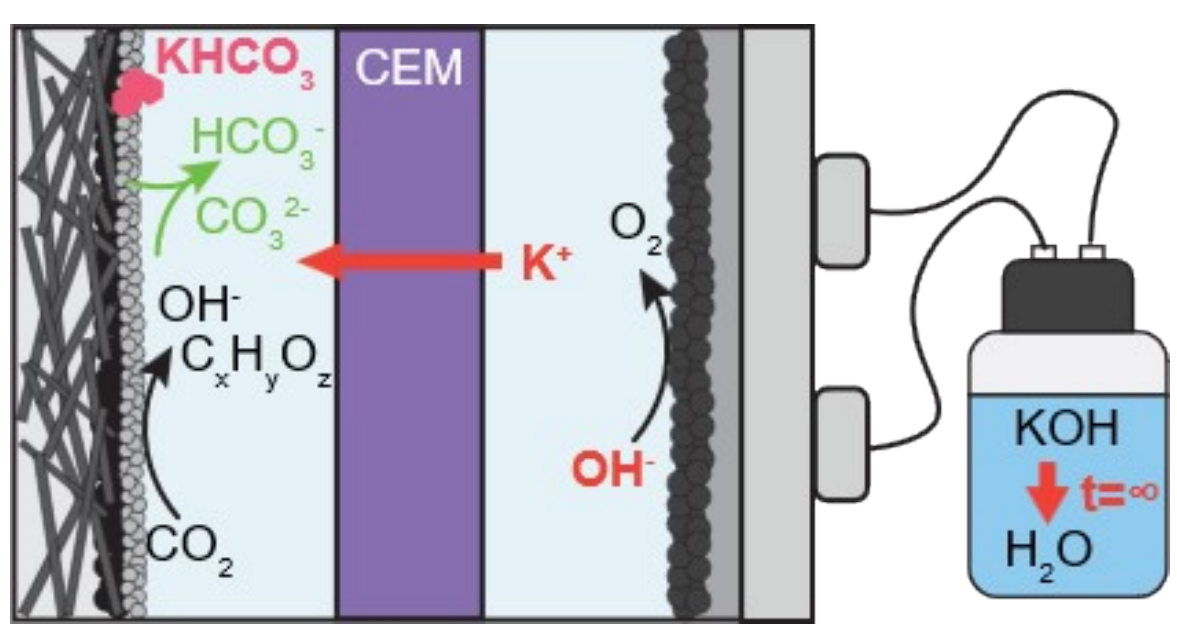

Fig. 7. Cation-exchange membrane CO_2 electrolyzer with alkaline anolyte (e.g: 1 M KOH). Over time the anolyte K^+ and OH^- will be depleted as K^+ will act as main charge carrier through the membrane and OH^- will be consumed by the oxygen evolution reaction (OER), converting the KOH anolyte (pH = 14) into H_2O (pH = 7). In a MEA cell configuration, the constant K^+ flux through the membrane will cause rapid cell failure due to salt precipitation at the cathode surface.

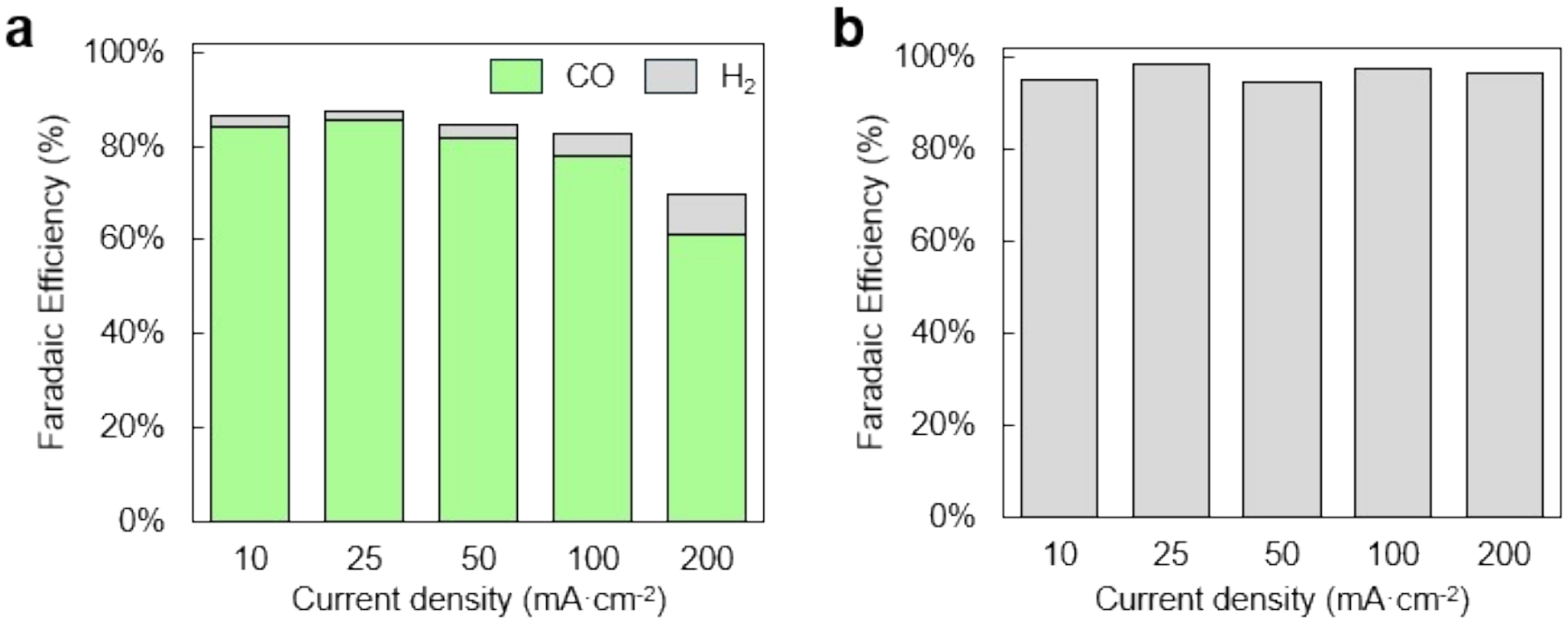

Fig. 8. Faradaic efficiencies for MEA CO_2 electrolysis experiments with different membrane configurations. a, AEM (Piperion 40 µm) and anolyte 1 M $KHCO_3$, **b,** CEM (Nafion 115) with anolyte 0.5 M H_2SO_4.

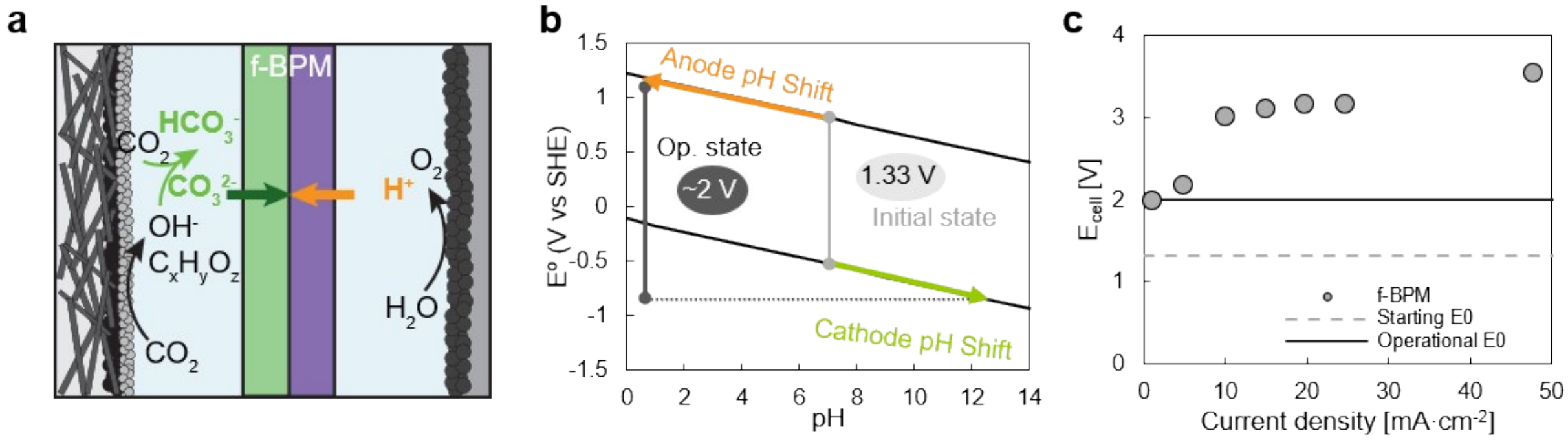

Fig. 9. Forward-bias (f-BPM) CO_2 electrolysis. a. Scheme of common phenomena in MEA f-BPM cells. At the cathode CO_2 is converted to carbonate and bicarbonate, while at the anode, pure water anolyte (pH = 7) transforms into H^+. Both H^+ and (bi)carbonates are combined at the interlayer of the BPM avoiding carbonate to cross to the anode. **b.** The local concentration polarization both at the anode and cathode leads to cell pH asymmetry and thus large concentration overpotential. **c.** Polarization curve of a f-BPMEA CO_2 electrolyzer.

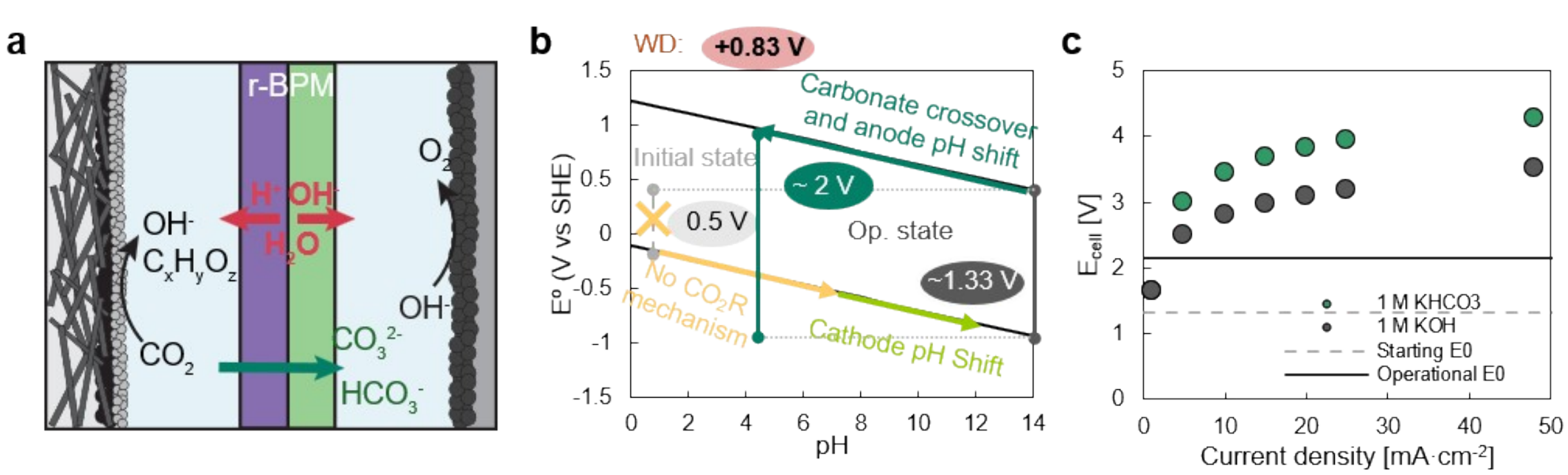

Fig. 10. Reverse-bias (r-BPM) CO_2 electrolysis. a. Scheme of common phenomena in MEA r-BPM cells. Water dissociation reaction takes place at the interface creating H^+ and OH^- that are transported towards cathode and anode respectively. Carbonate crossover can still occur in r-BPM leading to acidification of the anolyte. **b.** CO_2R cannot be performed under acidic conditions, so a pH shift is required at the cathode which will evolve into local alkaline

conditions. When OER is operated under alkaline conditions the operational cell voltage increases to ~2.16 V (1.33 V from electrode reactions + 0.83 V from water dissociation). If carbonate crossover through the BPM is considered, and thus, the anolyte pH shift to near-neutral conditions, the operational cell voltage can increase up to 2.83 V. **c.** Polarization curves of a r-BPMEA CO_2 electrolyzer with 1 M KOH and 1 M $KHCO_3$ anolyte.

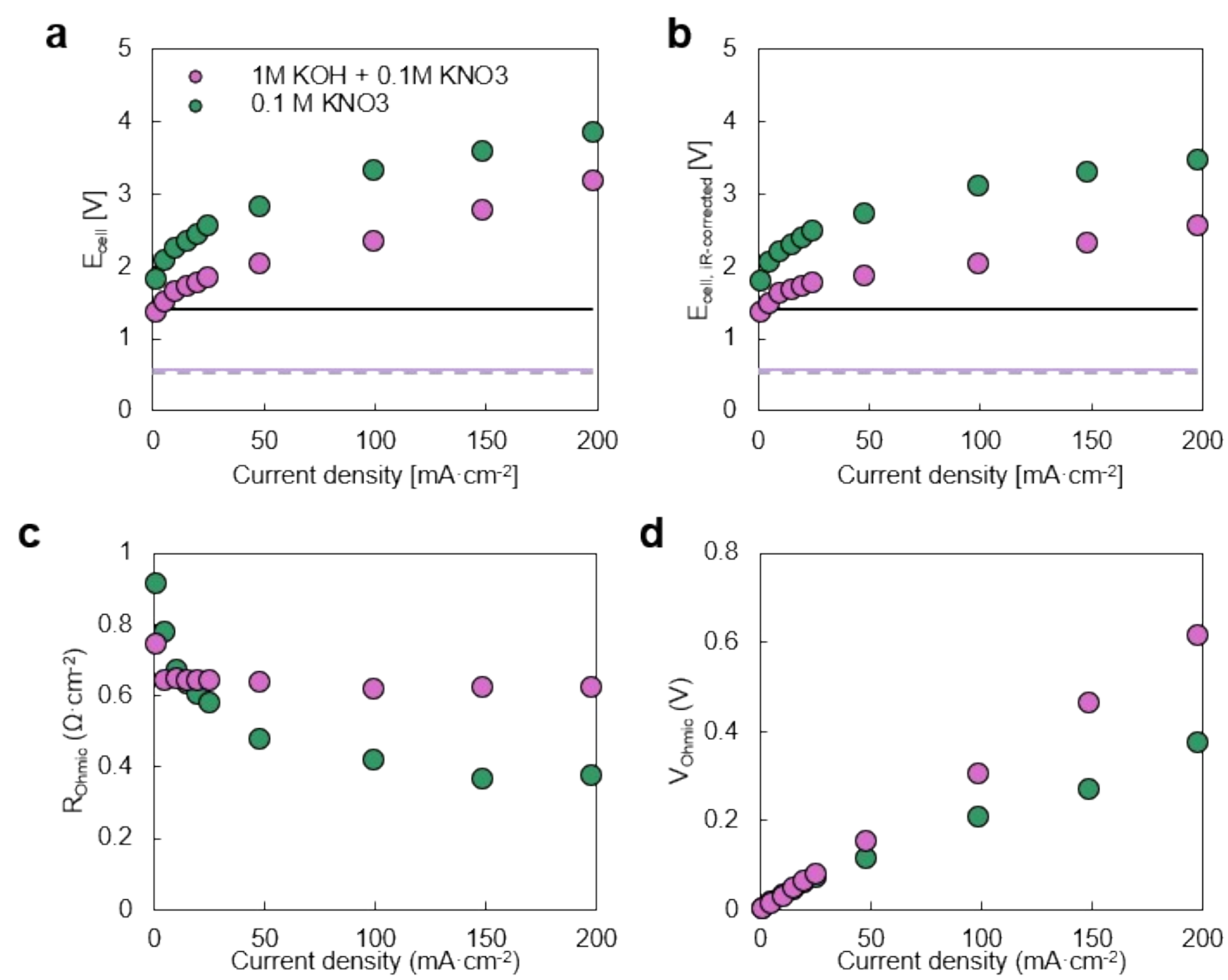

Fig. 11. Full polarization curves (1-200 mA·cm^{-2}) NO_3^- electrolysis (Fig. 4 in Manuscript). Polarization curves **a,** non-corrected and **b,** corrected for Ohmic drop. **c,** Ohmic resistance (Ω·cm^{-2}). **d,** Voltage penalty due to Ohmic resistance (V). Alkaline conditions: 0.1 M KNO_3 + 1 M KOH catholyte, 1 M KOH anolyte. Near-neutral conditions: 0.1 M KNO_3 catholyte, 1 M KNO_3 anolyte. In both scenarios a CEM was used.

Note: We expected higher Ohmic losses for the near-neutral pH scenario, although the concentration of the main charge carrier is 1 M K^+ for both cases. The differences observed can be due to compression or other Ohmic factors from cell assembly.

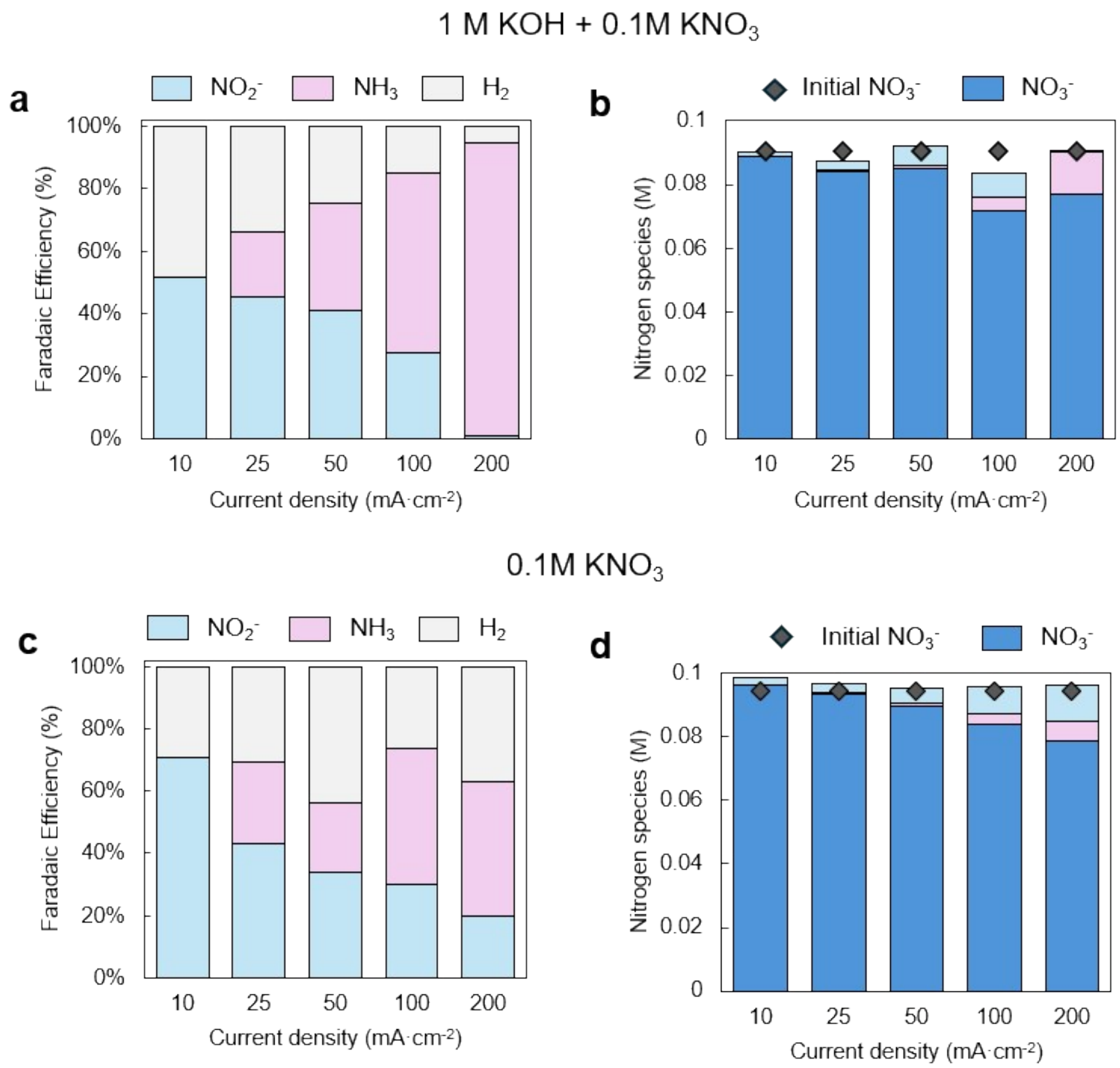

Fig. 12. Faradaic efficiencies and nitrogen balance in nitrate reduction experiments. Initial catholyte conditions: **a, b** 1 M KOH + 0.1 M KNO_3 and **c, d,** 0.1 M KNO_3.

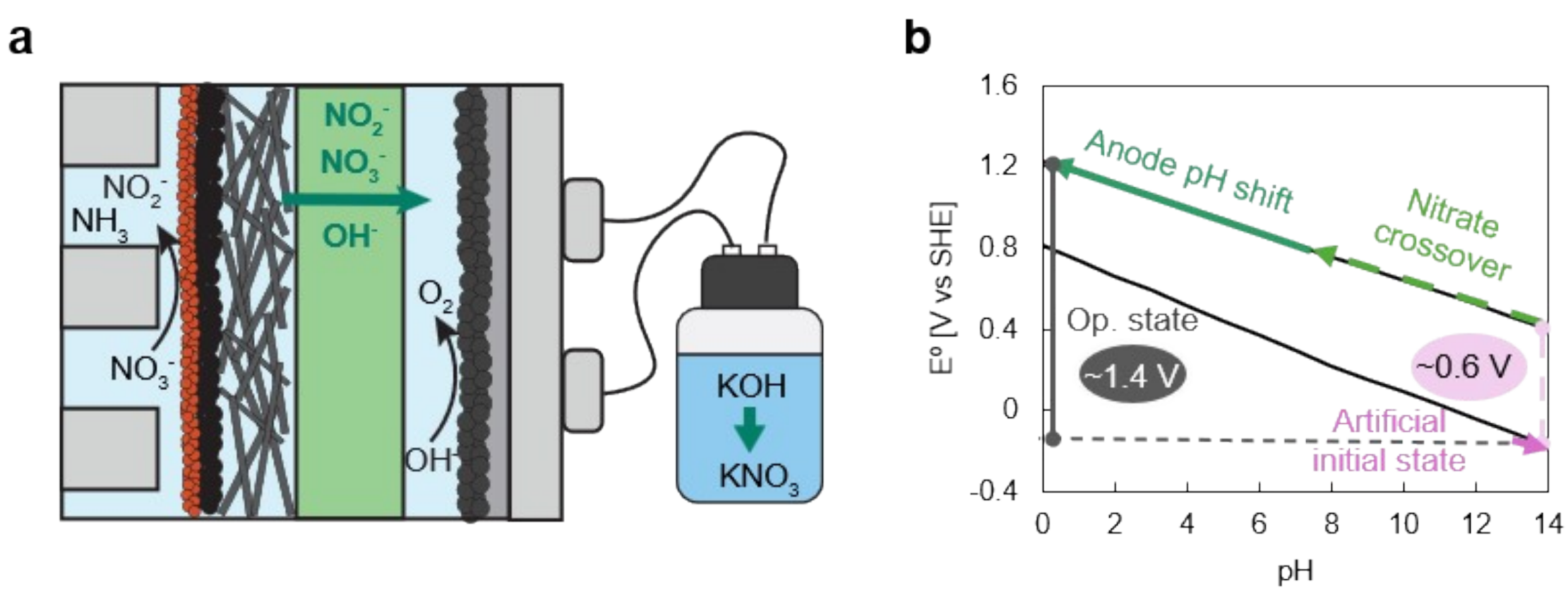

Fig. 13. Anion exchange membranes (AEM) in a nitrate MEA-electrolyzer. Due to the cationic nature of the AEM, negatively charged species can cross the membrane to the anode, leading to the acidification of the anolyte. The OH^- consumed by the OER at the anode are not replenished, and the KOH solution is slowly converted to KNO_3.

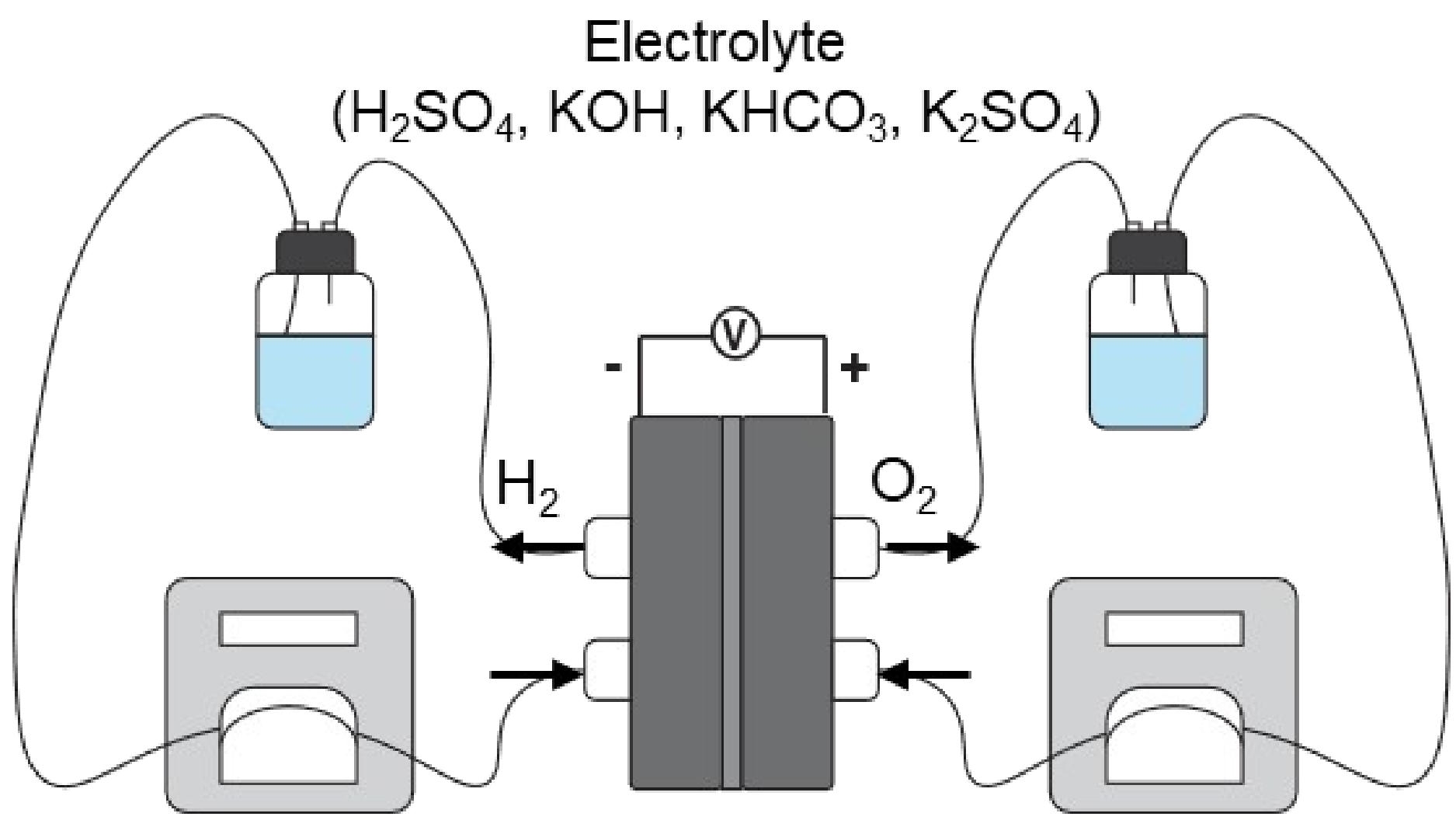

Fig. 14. Process flow diagram for water electrolysis experiments. MEA cell configuration. Catholyte and anolyte are recirculated at 20 ml·min^{-1}. Evolving H_2 and O_2 are released to exhaust.

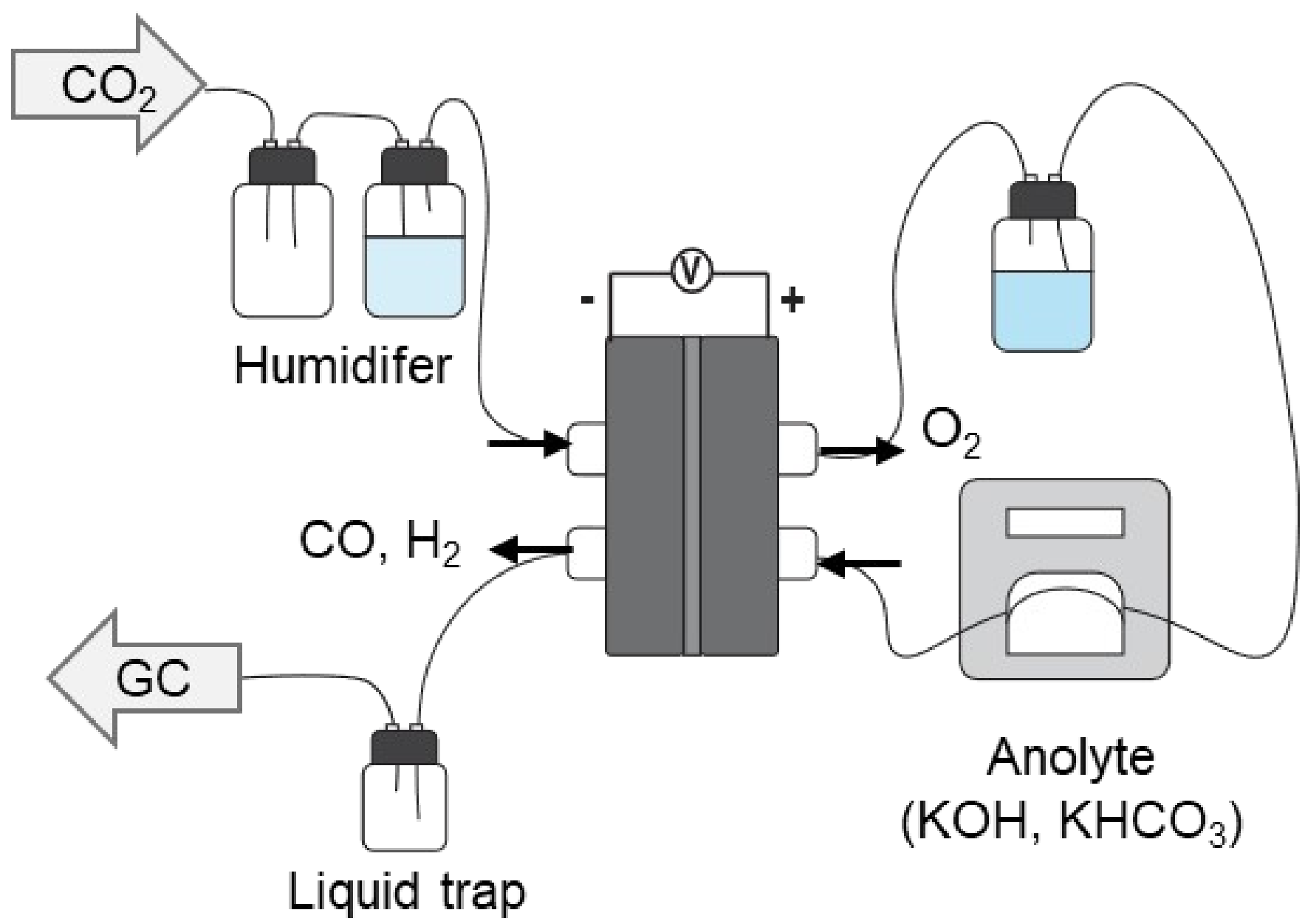

Fig. 15. Process flow diagram for CO_2 electrolysis experiments. MEA cell configuration. CO_2 is fed into a humidifier, and subsequently to the cathode inlet. The gas outlet is connected to the GC to analyze gas composition. At the anode side the anolyte is constantly recirculated at 20 ml·min^{-1}.

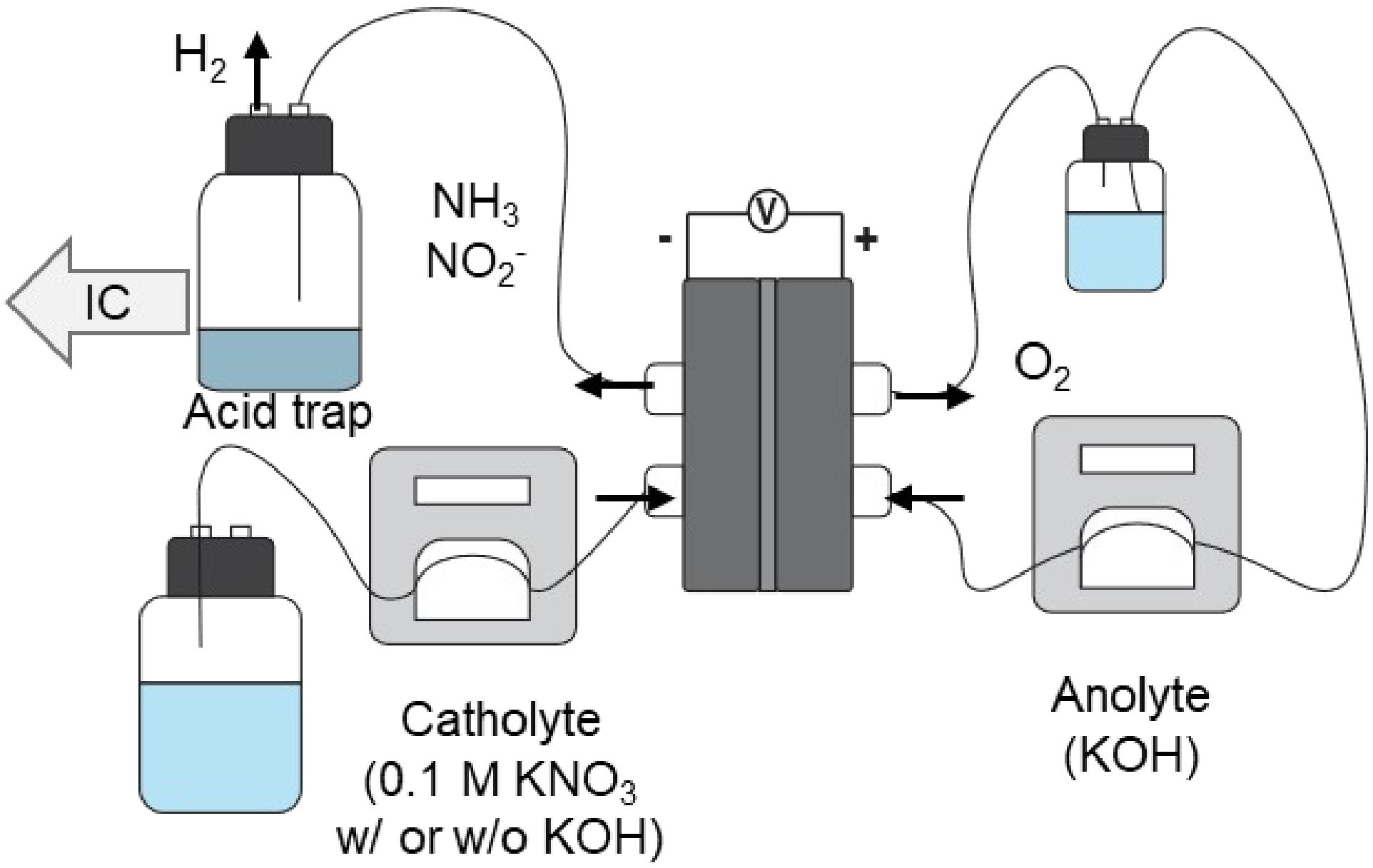

Fig. 16. Process flow diagram for nitrate electrolysis experiments. MEA cell configuration. The cathode side is operated in a single-pass configuration, in which 0.1M KNO_3 is fed (with or without additional electrolyte) at the cathode. The evolving species are collected in an acid trap (0.5 M H_2SO_4) to capture NH_3 in NH_4^+. The collected liquid is analyzed with ion-chromatography to determine the concentration of the products. On the contrary, the anolyte is constantly recirculated. The flowrates set by the peristaltic pumps is 5 ml·min^{-1}.

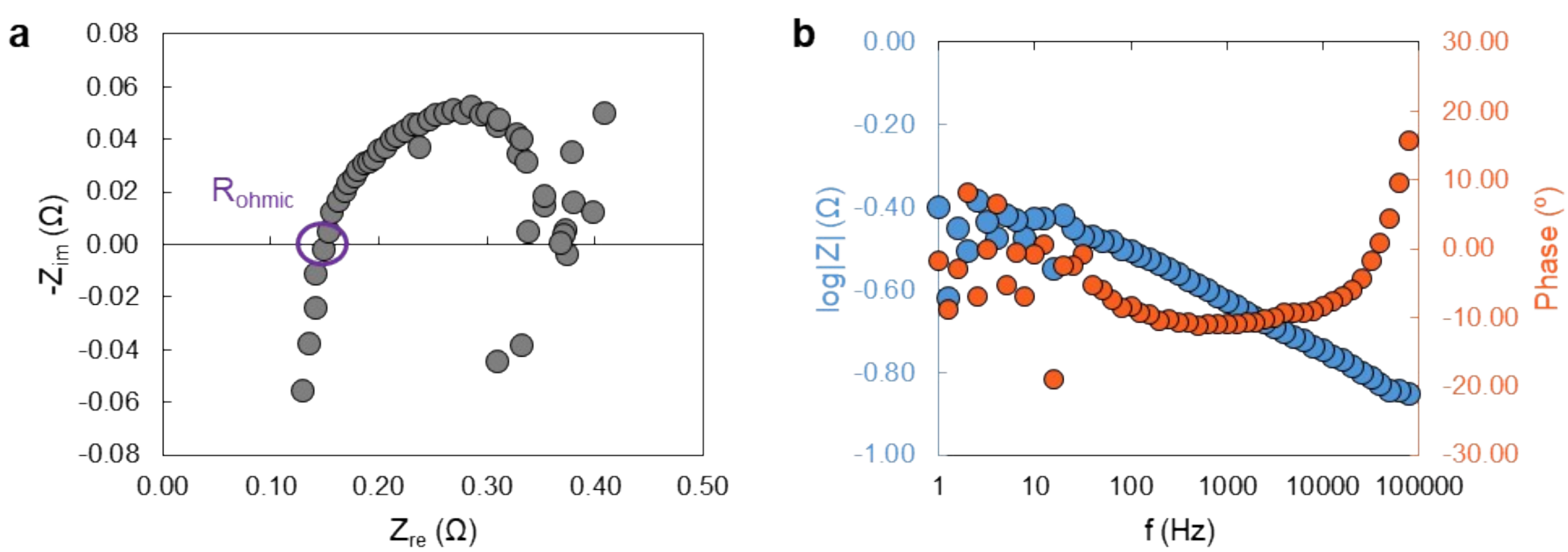

Fig. 17. Galvanostatic Electrochemical Impedance Spectroscopy. Example of **a.** Nyquist and **b.** Bode plot for a PEMWE at 100 mA·cm^{-2}. At lower frequencies noise leads to higher deviations. Ohmic resistance (R_{ohmic}) can be determined when Z_{im} or phase angle is zero.

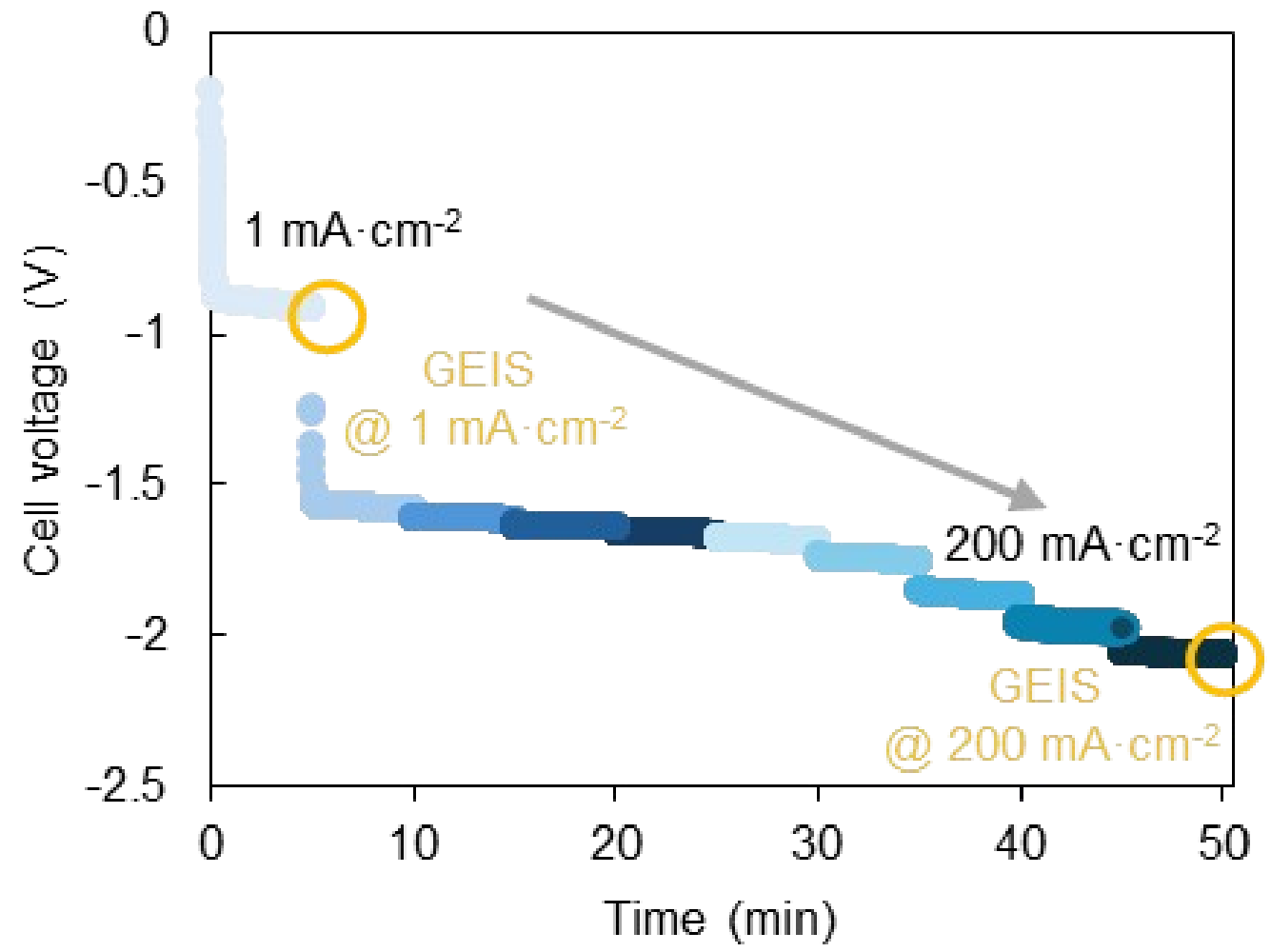

Fig. 18. Methodology to create polarization curves. Example of PEMWE experiment. Multiple steps at a constant current during 5 minutes, followed by Galvanostatic EIS measurement at the current density operated. When taking GC injections (CO_2 electrolysis) or samples for IC measurements (nitrate experiments) the step time was increased to 20 minutes.
